# Abrupt transformation of west Greenland lakes following compound climate extremes associated with atmospheric rivers

**DOI:** 10.1073/pnas.2413855122

**Published:** 2025-01-21

**Authors:** Jasmine E. Saros, Václava Hazuková, Robert M. Northington, Grayson P. Huston, Avery Lamb, Sean Birkel, Ryan Pereira, Guillaume Bourdin, Binbin Jiang, Suzanne McGowan

**Affiliations:** ^a^Climate Change Institute, University of Maine, Orono, ME 04469; ^b^School of Biology & Ecology, University of Maine, Orono, ME 04469; ^c^Department of Biology, Elizabethtown College, Elizabethtown, PA 17022; ^d^Cooperative Extension, University of Maine, Orono, ME 04469; ^e^The Lyell Centre, Heriot-Watt University, Edinburgh EH14 4AS, United Kingdom; ^f^School of Marine Sciences, University of Maine, Orono, ME 04469; ^g^School of Mechanical and Energy Engineering, Zhejiang University of Science and Technology, Hangzhou 310000, People’s Republic of China; ^h^Anji-Zhejiang University of Science and Technology Research Institute, Huzhou 313000, People’s Republic of China; ^i^Department of Aquatic Ecology, Netherlands Institute of Ecology, Wageningen 6708 PB, The Netherlands

**Keywords:** Arctic, ecosystem transformation, lake browning

## Abstract

Arctic ecosystems are affected by accelerated warming as well as the intensification of the hydrologic cycle, yet understanding of the impacts of compound climate extremes (e.g., simultaneous extreme heat and rainfall) remains limited, despite their high potential to alter ecosystems. Here, we show that the aquatic ecosystems in historically arid west Greenland have undergone an ecological transformation after a series of atmospheric rivers that simultaneously produced record heat and rainfall hit the region in autumn 2022. We analyzed a unique, long-term lake dataset and found that compound climate extremes pushed Arctic lakes across a tipping point. As terrestrial–aquatic linkages were strengthened, lakes synchronously transformed from “blue” lakes with high transparency and low pelagic primary production to “brown” in less than a year, owing to a large influx of dissolved organic material and metals, with iron concentrations increasing by more than two orders of magnitude. The browning of lake waters reduced light penetration by 50% across lakes. The resulting light limitation altered plankton distributions and community structure, including a major reduction in prokaryotic diversity and an increase in algal groups capable of metabolizing organic carbon sources. As a result, lakes shifted from being summer carbon sinks to sources, with a >350% increase in carbon dioxide flux from lakes to the atmosphere. The remarkably rapid, coherent transformation of these Arctic ecosystems underscores the synergistic and unpredictable impacts of compound extreme events and the importance of their seasonal timing, especially in regions with negative moisture balance.

## Significance

Arctic lake ecosystems are sites of high biodiversity that play an important role in carbon cycling, yet the impacts of emerging warmer and wetter conditions on the ecology of these lakes are poorly understood, partly owing to insufficient long-term data. Using a 10-y dataset, we report on an abrupt, coherent, climate-driven transformation of Arctic lakes in Greenland, demonstrating how a season of both record heat and rainfall drove a state change in these systems. This change from “blue” to “brown” lake states altered numerous physical, chemical, and biological lake features. The coherent lake state changes quantified here are unprecedented and may portend changes that can be anticipated more broadly in Arctic lakes as the hydrological cycle continues to intensify.

A global threat of climate change is ecosystem transformation (1, 2), involving system-level conversions such as biome (e.g., from shrubland to grassland) or ecosystem state transitions. The frequency of ecosystem transformations is increasing and predicted to accelerate (1, 3), with implications for biodiversity and ecosystem function including carbon cycling and sequestration (e.g., refs. 4 and 5). Ecosystem transformations may be driven by relatively gradual or abrupt climate change, with the role of climate extremes and interactions among multiple factors increasingly recognized as important drivers (6, 7).

Compound climate extremes are extreme events involving more than one variable, such as concurrent heat waves and droughts (8, 9). The compound nature of these events can cause unexpected impacts on human and natural systems (e.g., refs. 10 and 11). In mid- to high-latitude systems, atmospheric rivers (ARs, a long, narrow region of the atmosphere that transports water vapor) can produce compound climate extremes, as they can simultaneously transport intense moisture and heat poleward in synoptic-scale events. ARs affect many areas of the world, including western North and South America, western Europe, eastern United States, east Asia, and polar regions. In some areas, their frequency has increased over the past century (12), with unprecedented activity on the west coast of North America over the past decade (13). The scale of impacts when ARs make landfall depends on the hazard—including the intensity of moisture and heat transport, as well as the duration, frequency, and timing of events—and the vulnerability of the affected area—including antecedent conditions (e.g., soil moisture), topography, land use, and ecosystem type (14, 15). Models predict anywhere from a 50 to 290% increase in AR frequency in western North America, western Europe, east Asia, Greenland, and Antarctica by the end of this century (12), underscoring the need to better understand the ecological impacts of these compound extreme events and their potential to transform ecosystems.

While understanding of the impacts of compound extreme events on terrestrial and human systems has advanced over the past decade (e.g., refs. 14 and 16), much less is understood about how the impacts on terrestrial systems cascade to freshwaters, including lakes. With the vital ecosystem services provided by lakes, including freshwater storage on the landscape, drinking water, hydropower, habitat for rich biodiversity, and carbon sequestration, it is imperative to better understand the threat of compound extreme events such as ARs on these systems. Lakes are likely highly vulnerable to compound extreme events, given their sensitivity to univariate extreme events such as extreme precipitation (17) or heat (18). Even when univariate extreme events are short in duration, their impacts on lake ecosystems may persist by pushing the ecosystem over a threshold (19).

A fundamental classification system in lake ecology that describes ecosystem state is the nutrient-color paradigm (20), which predicts lake ecosystem production based on both nutrient concentrations [as total phosphorus (TP)] and water color owing to dissolved organic matter (measured as the absorbance at 455 nm), as these two parameters better reflect resource availability to producers than nutrients alone. This paradigm indicates that clear [low color, <20 platinum cobalt units (PCU)], low to moderate nutrient concentration (<30 mg/L TP) lakes are low productivity and referred to as “blue,” while lakes with similar nutrient concentrations but high color (>20 PCU) are “brown” Lakes with low color and high TP are “green” due to increased capacity for phototrophic productivity; lakes with both high color and high nutrients are “murky.” This classification system provides insight into the balance of autotrophy and heterotrophy in lake systems and is somewhat analogous to the “green” (plant-based) and brown (decomposer-based) food web concept used to describe terrestrial ecosystems (21). Changes in the balance of trophic state have implications for lake ecosystem structure (e.g., biodiversity) and function (e.g., carbon cycling, food web interactions) and hence ecosystem services (e.g., carbon burial). A strength of the nutrient-color framework is that transitions in lake ecosystem states can be detected fairly quickly and over relatively large spatial scales because it employs two commonly measured variables (e.g., ref. 22).

Lakes are important features of the Arctic landscape and are sites of intense mineralization of terrestrially derived organic carbon (23). With stronger warming at high latitudes, the >3.5 million lakes are particularly at risk of ecosystem transformation (6, 24). Aquatic systems, especially those affected by permafrost thaw, are substantial sources of greenhouse gas emissions (GHG) to the atmosphere (25), emitting more GHGs compared to the terrestrial landscape in which they sit. However, Arctic lakes lie at a sensitive threshold for shifts in metabolic balance (26), suggesting that there are many factors that likely lead to a higher degree of spatial and temporal variability in net auto- vs. heterotrophic states than is currently recognized. Hydrologic connectivity (defined as water-mediated transfer of materials to a lake from its landscape) has recently been identified as a key feature controlling the transport of carbon to freshwaters (27, 28). The strength of hydrologic connectivity varies across the Arctic, with lakes in more arid regions being CO_2_ sinks, at least during the summer (29). As the responses between climate, terrestrial–aquatic linkages, and carbon dynamics are tightly coupled, climate-driven shifts in hydrologic connectivity of lakes will propagate changing GHG emissions.

**Fig. 1.**
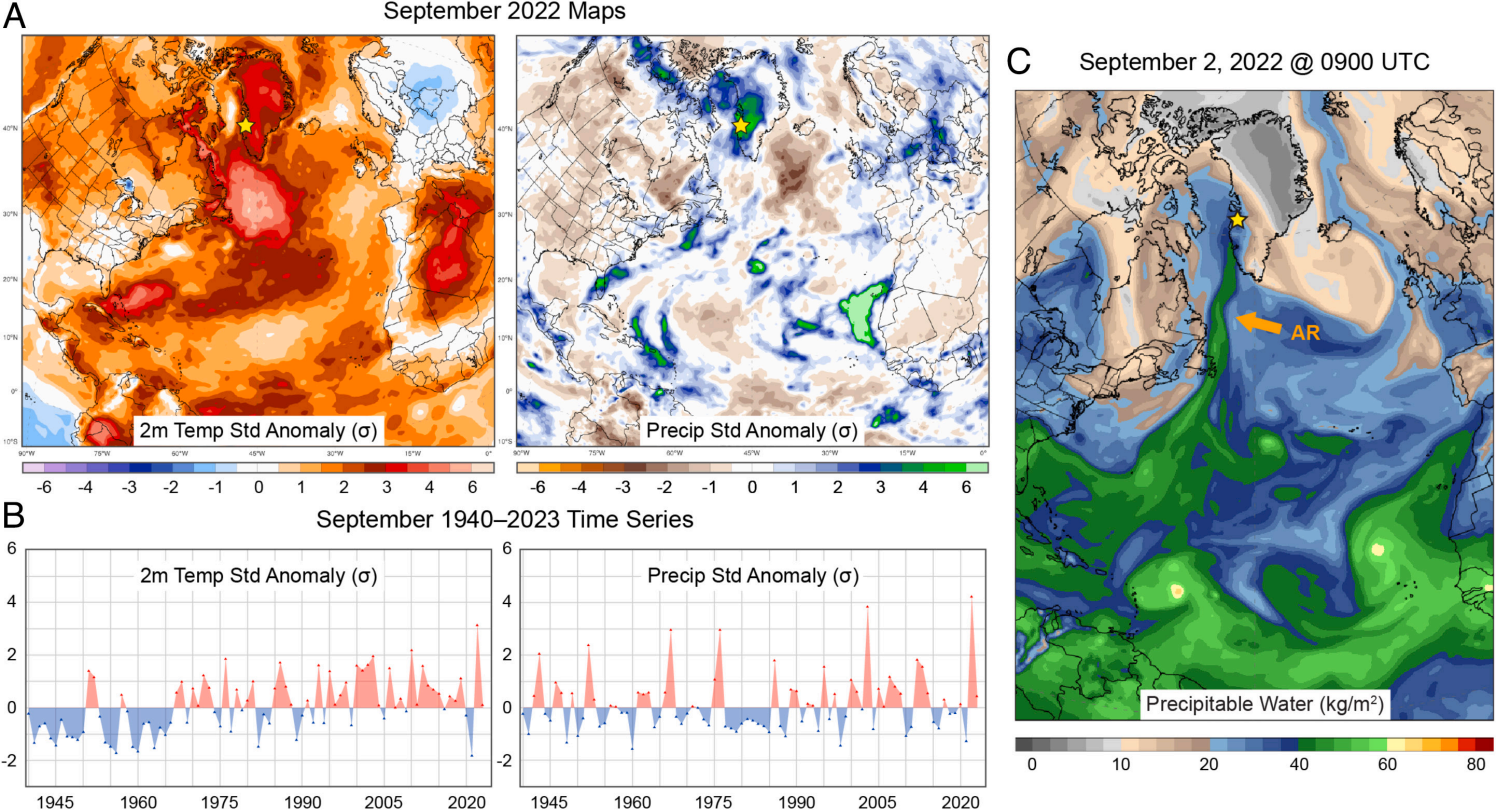
(*A*) Maps of 2-meter air temperature (2 m temp) and precipitation (precip) standardized anomalies for September 2022. (*B*) Time series of 2 m temp and precip standardized anomalies for September 1940 to 2023. (*C*) Map of precipitable water for September 2, 2022, at 0900 UTC, shown as an example of an AR (labeled AR) event. All anomalies are relative to 1951 to 2000 climatology. The study location is marked with a yellow star. Dataset: ECMWF Reanalysis Version 5 (ERA5) (31), processed using ClimateReanalyzer.org.

We quantified numerous biogeochemical, physical, and biological features of a suite of lakes in west Greenland (*SI Appendix*, Fig. S1) before and after a series of compound extreme events (record heat and precipitation) associated with ARs. According to the European Reanalysis version 5 (ERA5) data product, September 2022 was the warmest and wettest September recorded in west Greenland since 1940 (Fig. 1). The extremes of that month were driven by a series of ARs, beginning with a major event September 1 to 3 with total column precipitable water anomalies exceeding +5 σ from 1951 to 2000 climatology (*SI Appendix*, Fig. S2). This AR event was also associated with unusually warm air temperatures 3 to 4 σ above climatology that caused record late-season surface melt across the Greenland Ice Sheet (30). Other AR events impacted the region September 5, 11, 15 to 18, 23 to 24, and 25 to 26 (Movie S1). Importantly, observed minimum temperature at Kangerlussuaq remained above freezing for all but 3 d (*SI Appendix*, Fig. S3), meaning the majority of this precipitation fell as rain rather than snow. The latter event, September 25 to 26, was associated with the remnants of Hurricane Fiona and brought warm temperatures and record melting of the Greenland Ice Sheet for late September (30). Then, following a lull in storm activity, ARs again delivered moisture to west Greenland on October 16 to 19, 21 to 22, and 26 to 29 (Movie S2). ARs were also associated with extremes in west Greenland in July 2023, which reanalysis indicates was tied for the wettest and ranked among the warmest Julys since 1940 (*SI Appendix*, Fig. S4). The monthly mean total column precipitable water was nearly +5 σ above 1951 to 2000 climatology. On July 7 there was a major warm wave associated with an AR in which temperatures rose above freezing across almost half of the Greenland Ice Sheet (*SI Appendix*, Fig. S4*C*). Other AR events impacted the region July 9 to 11, 14, and 18 to 21 (Movie S3). Remote sensing suggests that an extensive and spatially heterogeneous area of the west Greenland landscape was affected by these storms, with the wetter soil conditions resulting collectively from the autumn 2022 and summer 2023 precipitation leading to a mosaic of terrestrial greening as indicated by higher Normalized Difference Vegetation Index (*SI Appendix*, Fig. S5). An estimated 7,486 lakes lie in the affected terrestrial area.

## Results

### Altered Regional Hydrology and Strengthened Terrestrial–Aquatic Linkages.

**Fig. 2.**
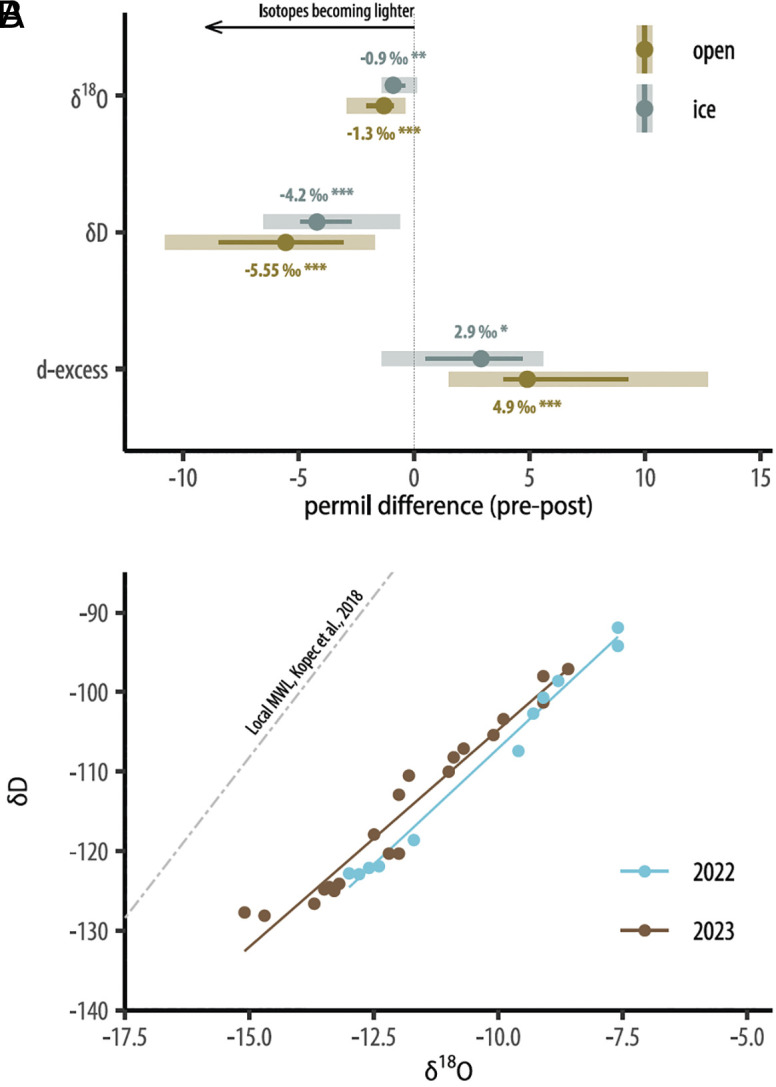
Shift in water stable isotopes before and after ecosystem transformation. (*A*) shows a decline in δ^18^O and δD and increase in d-excess under ice (April) and during summer (July and August) between 2022 and 2023. The central point is the median pre–post difference across lakes, the line shows the interquartile range, and the shaded region extends between maximum and minimum values. (*B*) shows the shift in the composition of δ^18^O and δD composition of lakes between summer 2022 (blue) and 2023 (brown). The dashed line shows the Local Meteoric Water Line (LMWL) based on data from ref. 34. Stars show levels of significance between pre- and postconditions: ***(*P* ≤ 0.001), **(*P* ≤ 0.01), *(*P* ≤ 0.05),^.^ (*P* ≤ 0.1), *ns* = not significant, *P* > 0.1.

The stable isotope composition of lake waters revealed that a hydrologic shift occurred between August 2022 (part of the 2022 summer ice-free season) and April 2023 (under ice), with δ^18^O values across lakes depleted by 4.8‰, δD values depleted by 0.9‰, and deuterium excess (d-excess) increased by 2.9‰ (Fig. 2*A*). Isotopic signatures from the previous ice-free season are preserved under ice (32), suggesting a large influx of isotopically depleted water and possibly reduced evaporation in September and October 2022 prior to ice formation in November, consistent with greater precipitation and cloud cover during those autumn months. Rising d-excess also reflects enhanced rainfall-generated runoff in permafrost environments (33). Both isotopes were further depleted after ice out in 2023, pushing closer to meteoric water composition (Fig. 2*B*) and reflecting reduced evaporation, likely owing to the above average cloud cover in May and June 2023 (*SI Appendix*, Fig. S6).

**Fig. 3.**
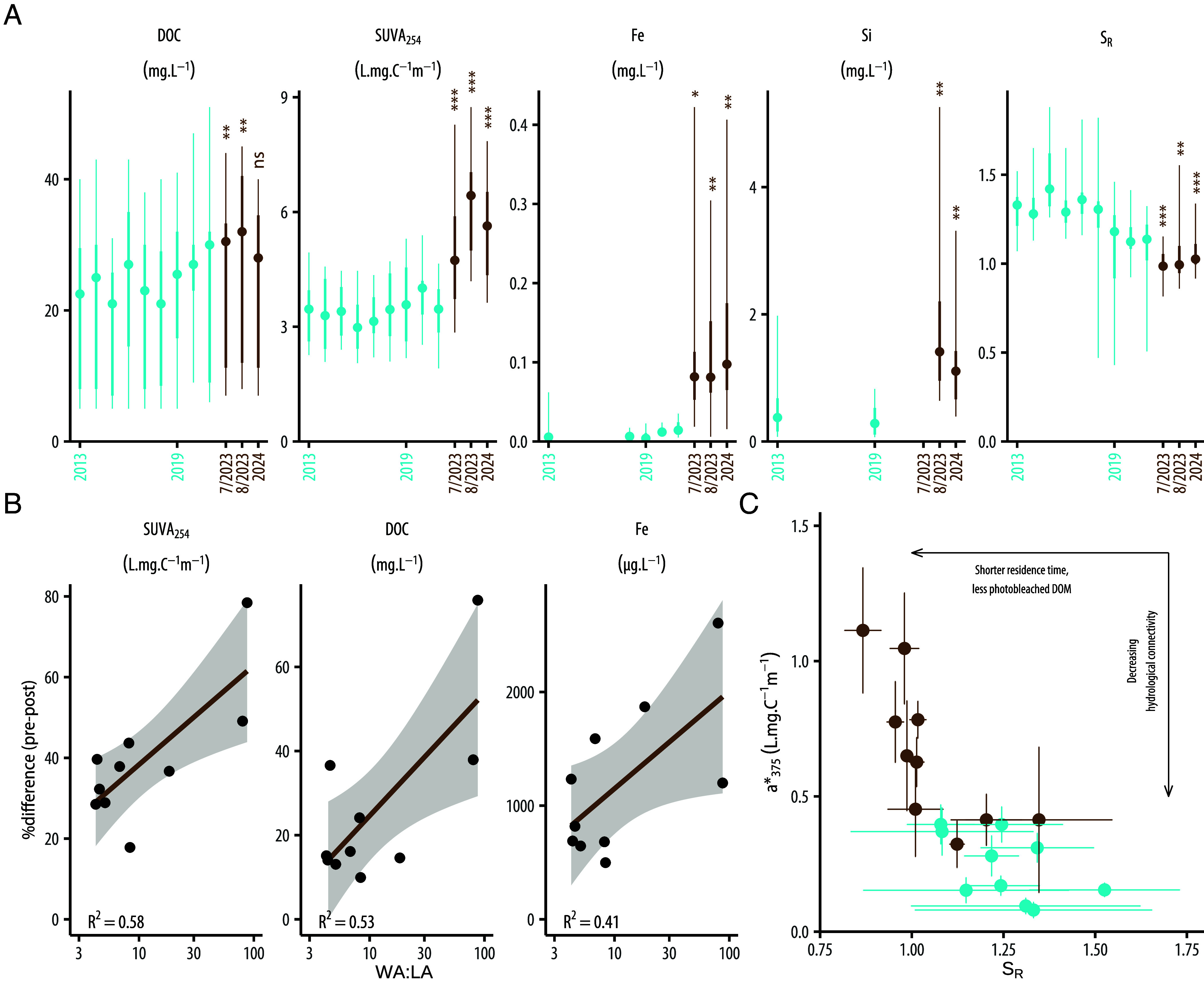
Intensified hydrological connectivity led to higher inputs of terrestrial material into aquatic systems. (*A*) shows a marked shift in epilimnetic DOC concentration, SUVA_254_, iron, silica, and SR in July and August 2023 and July 2024 (brown) compared to summer data (June to August) since 2013 (blue). The central point is the median value across lakes, the thicker line depicts the interquartile range, and the thinner line shows maximum and minimum values. (*B*) Pre–post % change in June-July SUVA_254_, DOC, and Fe concentrations increases linearly with watershed to lake area ratio. Each black point represents the pre–post % difference in each lake. (*C*) shows the simultaneous decrease in SR and increase in a*_375_ in 2023 compared to previous years (2013 to 2022). Each point is a mean and error bars represent SD.

The altered hydrology of the area substantially increased the connectivity of lakes with their watersheds, thereby strengthening the transfer of terrestrially derived materials into aquatic systems that was evident in early July 2023. Average lakewater dissolvedorganic carbon (DOC) concentrations increased by 22% compared to 2013 to 2022 averages (t = 3.13, df = 9, *P* = 0.012; Fig. 3*A*), and the source and quality of this material abruptly shifted to a larger terrestrial component. Early July 2023 lakewater specific ultraviolet absorption at 254 nm (SUVA_254_), an index of aromatic carbon content, increased by 39% across lakes compared to 2013 to 2022 (t = 5.05, df = 9, *P* < 0.001), while the ratio of spectral slopes (S_R_), indicative of dissolved organic matter (DOM) quality, declined by 19% (t = −9.07, df = 9, *P* < 0.001) across lakes (Fig. 3*A*). Higher SUVA_254_ and lower S_R_ values indicate more soil-derived DOM and greater hydrologic connectivity (35). Larger increases in SUVA were found in lakes with larger watershed area to lake area (WA:LA; R^2^ = 0.58; Fig. 3*B*), consistent with the terrestrial origin of this material. The degree of DOM color, represented by a*_375_ (the ratio of the absorption at 375 nm to DOC concentration), was 131% higher in early July 2023 compared to all previous years (t = 5.08, df = 9, *P* < 0.001; Fig. 3 *A* and *C*), including data from 2002 & 2003 (*SI Appendix*, Fig. S7). Higher a*_375_ indicates greater input of terrestrial DOM and stronger hydrologic connectivity between watersheds and lakes (36); the high values in 2023 are more typical of coastal lakes in Greenland situated in a higher precipitation area. Analysis of DOM size fractions using liquid chromatography- organic carbon and nitrogen detection revealed increases in humic substances (Mr ca. 1,000 g/mol) and in biopolymers with a high molecular weight (>20,000 g/mol; *SI Appendix*, Fig. S8), further indicating a larger terrestrial contribution of DOM in 2023 compared to 2018. The proportions of carbon:nitrogen (C:N) in these DOM fractions indicate that DOM in 2023 was more N-depleted than in 2018 (*SI Appendix*, Table S1), suggesting a larger supply of polysaccharides and lignin-derived materials. Over the same time period, low molecular weight acids (<350 g/mol) declined which may indicate that either these acids are not transported to the lake or that utilization of these acid compounds is greater than the production. Together, these observations are consistent with a greater flux of recalcitrant DOM being delivered to the lakes, suggesting mobilization of old organic matter from the catchments to the lakes.

Total iron concentrations in water in early July 2023 increased by more than 1,000% across lakes (t = 2.9, df = 9, *P* = 0.018; Fig. 3*A*), with larger increases in lakes with larger WA:LA (R^2^ = 0.41; Fig. 3*B*). Concentrations of other metals (e.g., Al, Co, Cr, Cu) also increased across lakes (*SI Appendix*, Fig. S9). Dissolved silica in lakewater, which originates from weathering of watershed bedrock and has greater solubility than iron and aluminum–silicate–humic complexes, increased by 448% across lakes compared to concentrations in 2013 and 2019 (t = −3.95, df = 9, *P* = 0.003; Fig. 3*A*). Trends in gradually increasing iron and DOM have occurred broadly in northern hemisphere lakes over recent decades, with these increases attributed in part to increasing precipitation and subsequent changes in soil redox conditions (37). More frequent waterlogging of soils creates reducing conditions and subsequent release of iron and organic C (38, 39). With large stores of soil organic matter in permafrost and tight coupling of iron and carbon cycles (38, 40), climate-driven alterations to soil redox conditions have tremendous potential to release organic carbon into Arctic surface waters.

These changes in lakewater chemistry did not alter the acid neutralizing capacity across lakes (t = 0.94, df = 9, *P* = 0.37; *SI Appendix*, Fig. S10), with median values of 1,900 to 2,000 µeq L^−1^. In early July 2023, lakewater pH was lower, dropping from 8.01 to 7.88 across lakes (t = −5.49, df = 8, *P* < 0.001; *SI Appendix*, Fig. S10) but remaining circumneutral.

Further evidence of substantially altered terrestrial–aquatic linkages is the increase in nutrients across lakes by early July 2023 compared to 2013 to 2022 (*SI Appendix*, Fig. S11), including TP (66% increase; t = 4.23, df = 9, *P* = 0.002) as well as both forms of dissolved inorganic nitrogen (DIN) [nitrate (200% increase; t = 2.34, df = 9, *P* = 0.04) and ammonium (123% increase; t = 2.56, df = 9, *P* = 0.03)]. Under reducing conditions in permafrost soils, phosphate can be released from iron oxides into pore waters by microbial reduction (41), while permafrost thaw releases long-frozen stores of various forms of nitrogen, including inorganic nitrogen forms (42). Altered internal nutrient cycling in lakes as a source of these elevated nutrient concentrations is less likely since dissolved oxygen profiles of lakes were no different in 2023 compared to previous years (*SI Appendix*, Fig. S12). While TP and DIN concentrations increased, there were no changes in DIN:TP (t = 1.45, df = 9, *P* = 0.18), an indicator of nutrient limitation status in lakes.

Many of these changes in lake biogeochemistry have persisted and in some cases, increased further with the anomalously high July 2023 total precipitation. Over the summer of 2023, further increases in DOC concentration (15%), SUVA_254_ (40%), a*_375_ (170%), and TP (15%) were apparent by August (Fig. 3*A* and *SI Appendix*, Fig. S11). One exception was nitrate, which essentially declined back to pre-2023 concentrations, likely due to biological uptake during the growing season. Lakes were sampled again in early July 2024 and demonstrated sustained changes compared to pre-event conditions (Fig. 3*A*). In particular, DOC concentrations were 19.6% higher compared to preaverage (t = −1.61, df = 9, *P* = 0.14), Fe concentrations were 1,079% higher (t = −3.41, df = 9, *P* = 0.008), S_R_ was 14.9% lower (t = 5.23, df = 9, *P* < 0.001), SUVA_254_ was higher by 61.2% (t = −9.72, df = 9, *P* < 0.001), dissolved silica was 243% higher (t = −4.33, df = 9, *P* = 0.002) and a*_375_ was 195% higher (t = −7.96, df = 9, *P* < 0.001).

### Stronger Hydrologic Connectivity Transforms Lake Ecosystems.

**Fig. 4.**
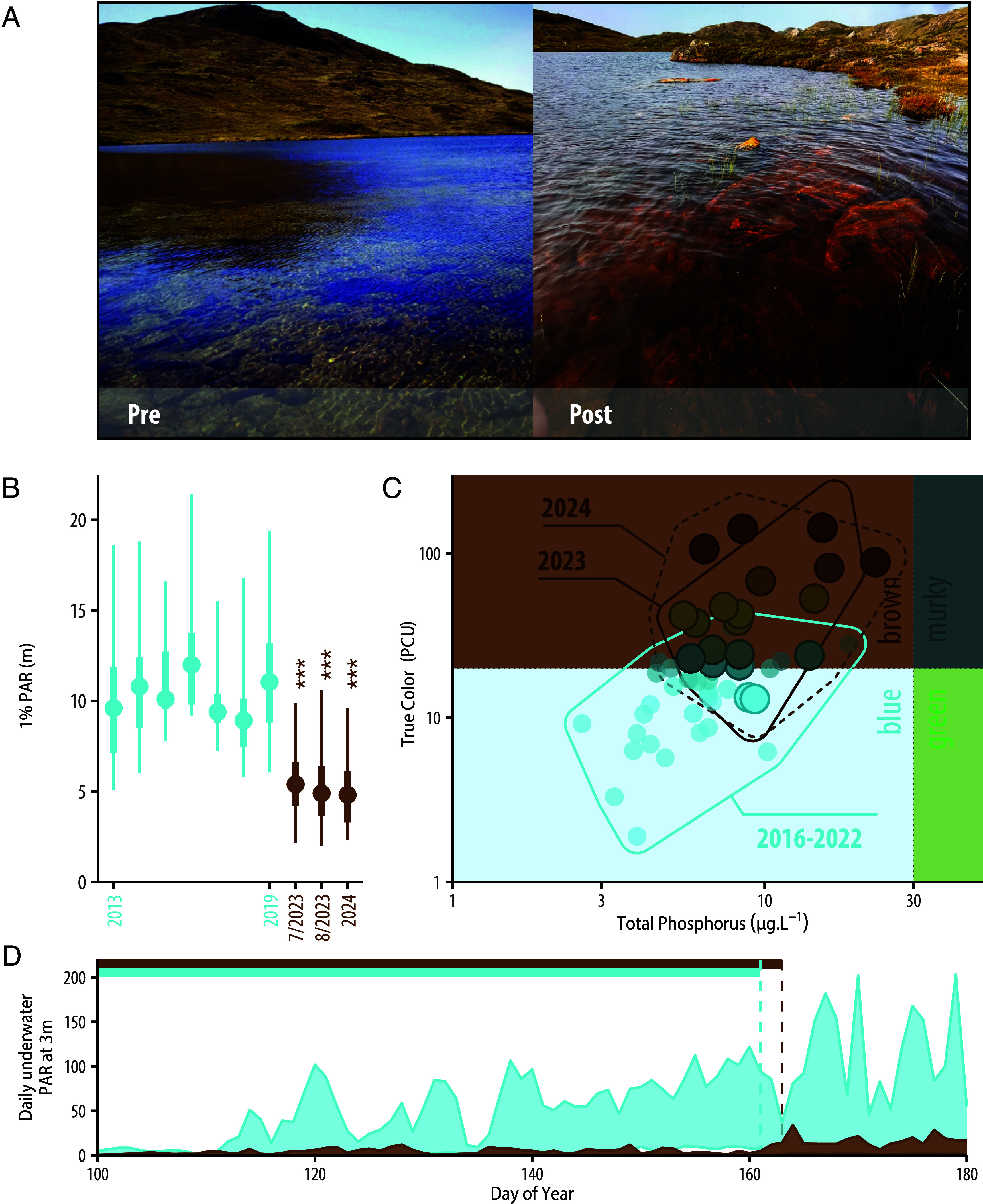
Transformation of lakes from *blue* to *brown*. (*A*) shows that clearwater, *blue*, lakes (photo from summer 2014) became darkly stained, *brown*, in 2023 (photo from summer 2023). (*B*) The central point is the median depth of 1% PAR across lakes for each year since 2013, the thicker line shows the interquartile range, and the thinner line shows maximum and minimum values. Blue distributions show data before 2023, and brown show data from July and August 2023 and July 2024. (*C*) Nutrient-color status of lakes shifted in July 2023 & 2024 compared to July of 2016 to 2022, with lakes moving toward the brown quartile. Both axes are on a log10 scale. (*D*) Light underwater was substantially reduced in 2023 (brown) compared to 2022 (blue) in lake SS85. The ribbons show the interquartile range of daily values from a PME miniPAR sensor anchored 3 m below the surface from mid-April (DOY 100) to the end of June (DOY 180). Bars on the *Top* of the panel show ice-cover, and vertical dotted lines represent the timing of ice-out during each year.

This influx of materials from the watershed strongly affected the physical features of lakes, transforming these ecosystems. The most striking visible change was the abrupt, coherent transition from waters with high clarity from 2013-2022 to those that are now darkly stained (Fig. 4*A*), likely owing to the changes in DOM quality and increased iron concentrations. The majority of lakes, which were categorized as blue from 2016 to 2022, were transformed to brown in less than a year, with a 190% increase in color across lakes (t = 3.02, df = 9, *P* = 0.013; Fig. 4*C*). The magnitude of color change in less than 1 y in these lakes was comparable to that which occurs in boreal lake ontogeny (the developmental history of lakes) over hundreds to thousands of years (43). This change in water color was sustained in July 2024 (t = −3.47, df = 9, *P* = 0.007; Fig. 4*C*). Water clarity, measured as the 1% attenuation depth of photosynthetically active radiation (1% PAR), was coherently reduced by 49% across lakes in summer 2023 compared to summers of 2013 to 2019 (t = 13.9, df = 9, *P* < 0.001; Fig. 4*B*); this change was also sustained in summer 2024 (t = 15.9, df = 9, *P* < 0.001). Based on in situ light sensors deployed year-round at a few meters below the lake surface and recording hourly, reduced water clarity was apparent even under the 2023 spring lake ice, and continued through the summer (Fig. 4*D*).

**Fig. 5.**
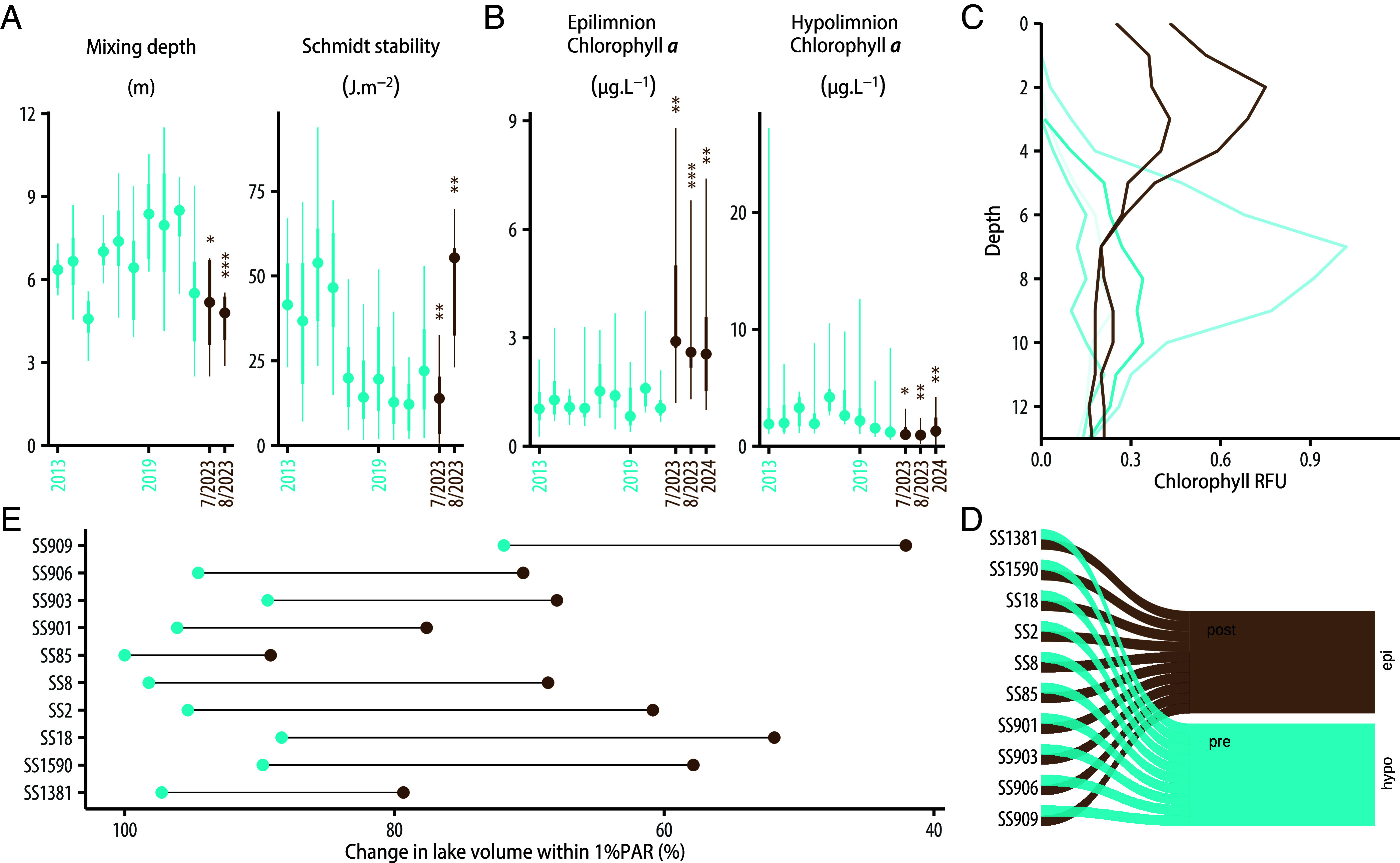
Changes in lake properties driven by reduction of transparency. (*A*) Time series of *A* lake thermal structure metrics: mixing depth and Schmidt stability, and (*B*) epilimnetic and hypolimnetic chlorophyll-a concentrations. The central point is the median value across lakes for each year since 2013, the thicker line shows the interquartile range, and the thinner line shows maximum and minimum values. Blue distributions show data from 2013 until 2022, brown distributions show data from July and August 2023 and July 2024 for chlorophyll data. (*C*) Vertical profiles of chlorophyll (relative fluorescence units, RFU) from lake SS909 showing vertical shift in peak chlorophyll from bottom waters in 2016 to 2022 (blue) to surface waters in 2023 (brown). (*D*) Chlorophyll-a maxima have moved from hypolimnion to epilimnion after the browning event in 2023. (*E*) The blue point represents the average lake volume within 1% PAR before 2023, and the brown point represents lake volume post browning in 2023.

Increased color and subsequent reduced water clarity altered the vertical structure and habitat distribution within lakes. In the summer, these lakes thermally stratify, with a warmer, mixed layer at the surface over a cooler, deep layer; variability in the thickness of the surface layer is strongly controlled by water clarity, with wind, the timing of ice melt, and air temperature also contributing (44, 45). Median surface layer thickness in August 2023 was 4.8 m, 31% shallower compared to previous years (t = −6.07, df = 6, *P* < 0.001), and stratification as indicated by Schmidt’s Stability Index was 68% stronger and more stable (t = 3.46, df = 6, *P* = 0.014; Fig. 5*A*). Shallower, stronger stratification was likely owing to the increased color of waters, as mean summer air temperatures were lower than average (June-July-August mean of 9 °C in 2023 compared to 10 °C for 1975 to 2021) and ice melt occurred fairly late (~June 15); both factors would drive deeper, weaker stratification in this area (44, 45). Vertical habitat gradients changed, with the peak in water column concentrations of chlorophyll *a*, a measure of total phytoplankton biomass which serves as the “green” base of pelagic food webs, shifting from deeper to shallower waters across all lakes in 2023 compared to all previous years (Fig. 5 *B*–*D*). Compared to previous years, early July 2023 surface layer chlorophyll *a* across lakes increased by 217% (t = 4.08, df = 9, *P* = 0.003) while deep layer chlorophyll *a* declined by 43% (t = −2.69, df = 9, *P* = 0.025). These changes were sustained in July 2024 (Fig. 5*B*), with epilimnion values 165% higher (t = −3.35, df = 9, *P* = 0.009) and hypolimnion values 54.1% lower (t = 3.33, df = 9, *P* = 0.009) compared to 2013 to 2022. The depth of the chlorophyll *a* maximum often tracks changes in the 1% PAR depth, as light is a key resource for phytoplankton. Across lakes, the volume of the photic zone (where at least 1% PAR is available to support photosynthesis) declined across lakes by 28% (t = −9.72, df = 9, *P* < 0.001; Fig. 5*E*). While the vertical distribution of chlorophyll *a* and total volume of the photic zone changed, integrated total water column chlorophyll *a* did not change in 2023 (t = 1.07; df = 9; *P* = 0.31; *SI Appendix*, Fig. S13).

**Fig. 6.**
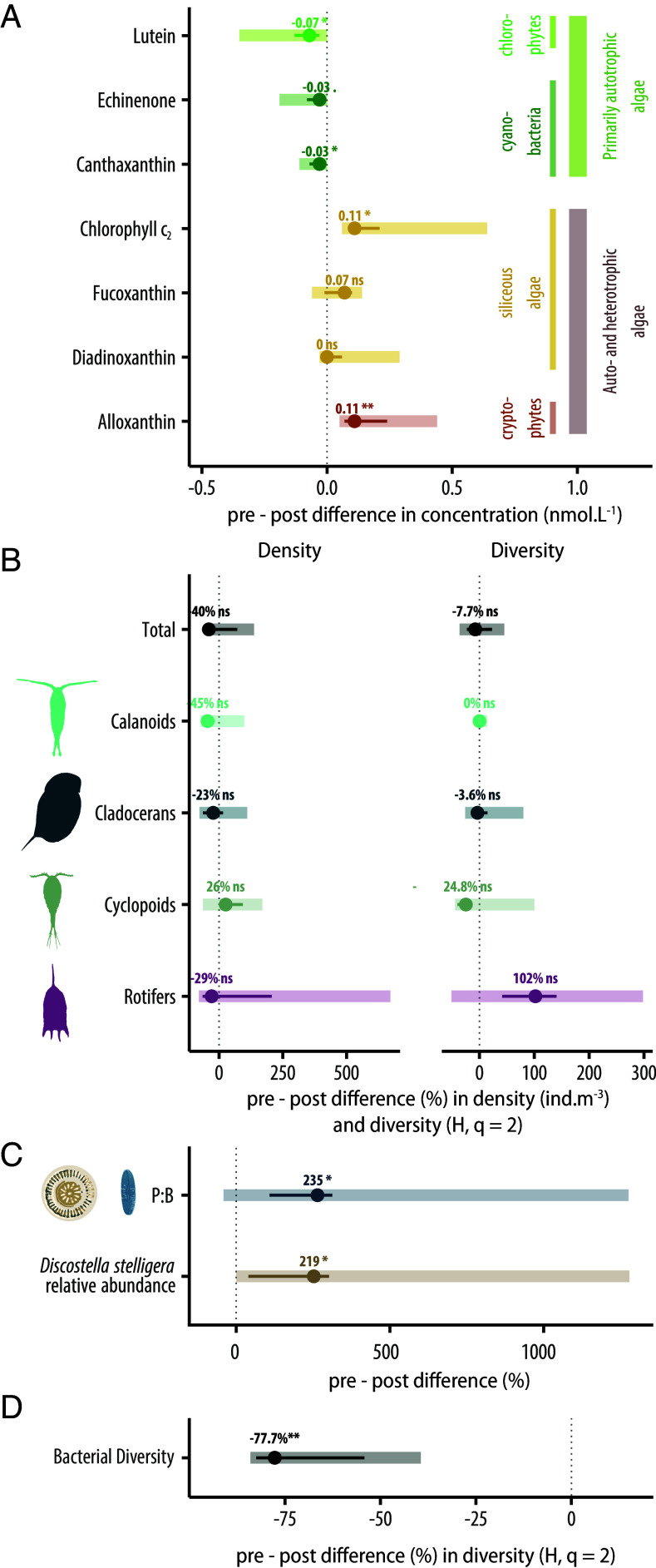
Ecological transformation in lakes post browning event in 2023. (*A*) Photosynthetic pigments from epilimnetic water samples (2010 to 2011 vs. 2023), (*B*) zooplankton density and diversity across major taxonomic groups (2014 vs. 2023), (*C*) diatoms from surface sediments (2013 vs. 2023), and (*D*) bacterial communities (2015 and 2019 vs. 2023). The central point is the median pre–post value across lakes, the line shows the interquartile range, and the shaded region extends between maximum and minimum values.

These changes in lake physical structure and biogeochemistry altered plankton community structure and diversity. Photosynthetic pigments in lake surface mixed layers revealed an increase in alloxanthin (indicative of cryptophytes), fucoxanthin (indicative of diatoms and chrysophytes), and chlorophyll c_2_ (indicative of these same groups plus dinoflagellates) across lakes in August 2023 compared to 2010 (Fig. 6*A*; (46)). These pigment changes suggest an increase in the proportion of mixotrophic taxa- those that can combine auto- and heterotrophy, feeding on organic carbon sources and bacterial communities- compared to 2010, when pigments of primarily autotrophic groups [e.g., echinenone and canthaxanthin (indicative of cyanobacteria), lutein (indicative of chlorophytes)] were more abundant. Pigment analyses of deepwater surface sediments, which integrate algal remains over many months, reflected similar shifts in the phytoplankton communities (*SI Appendix*, Fig. S14; (46)). While total zooplankton diversity did not change in 2023 compared to 2014 (t = −0.403, df = 5, *P* = 0.70; Fig. 6*B*; (46)), diversity of cyclopoid copepods decreased (t = −0.88, df = 5, *P* = 0.46) and rotifer diversity increased (t = 1.67, df = 5, *P* = 0.16), although not significantly. Rotifer communities in both years were dominated by microphagous (i.e., feeding on microorganisms) taxa (t = −0.20, df = 5, *P* = 0.86; *SI Appendix*, Table S2). While changes in zooplankton density were not significant across lakes (t = 0.18, df = 5, *P* = 0.86), mean total density was lower in 2023, with the only increase in density observed for cyclopoid copepods, which prey on rotifers. Increases in cyclopoid copepods and changes in rotifer communities were also observed with long-term (multidecadal) lake browning in two lakes in the northeastern US (47), with these changes in part attributed to altered water transparency and vertical habitat gradients. Overall, the ratio of planktonic consumer to producer (C:P) biomass did not change across lakes (t = −0.27; df = 5, *P* = 0.80; *SI Appendix*, Table S3); C:P was 3.6 in August 2014, and 4.1 in August 2023.

Comparing diatom assemblages in lake surface sediments collected in 2023 to those from 2013, the planktonic to benthic ratio (P:B) increased 235% in 2023 (t = 2.71, df = 8, *P* = 0.03; Fig. 6*C*; (46)), indicating a strong and widespread loss of benthic habitat across lakes owing to the observed light reduction. Consistent with the browning-induced thermal structure changes observed in these lakes, the percent relative abundance of *Discostella stelligera*, an indicator taxon of shallower lake mixing depths (48), increased by 219% across lakes (t = 2.36, df = 8, *P* = 0.05; Fig. 6*C*).

Bacterial diversity declined with the transition to brown lakes (Hill number of order q = 2; t = 4.9921, df = 6.2138, *P* = 0.002235; Fig. 6*D*; (46)). Environmental DNA of the upper mixed layer identified a total of 194 bacterial taxa across all lakes and sample years. The number of taxa identified declined by 90% in 2023, with 191 taxa detected across all lakes in samples collected prior to 2023, and 20 identified across the same sites in 2023. Three taxa were unique to 2023, including *Nostoc PCC-73102*, *Methanobacterium,* and *Gemmata*. Similar declines in bacterial diversity with brownification have also been observed in freshwater mesocosm experiments (49).

### Implications of Lake Ecosystem Transformation for Carbon Cycling.

**Fig. 7.**
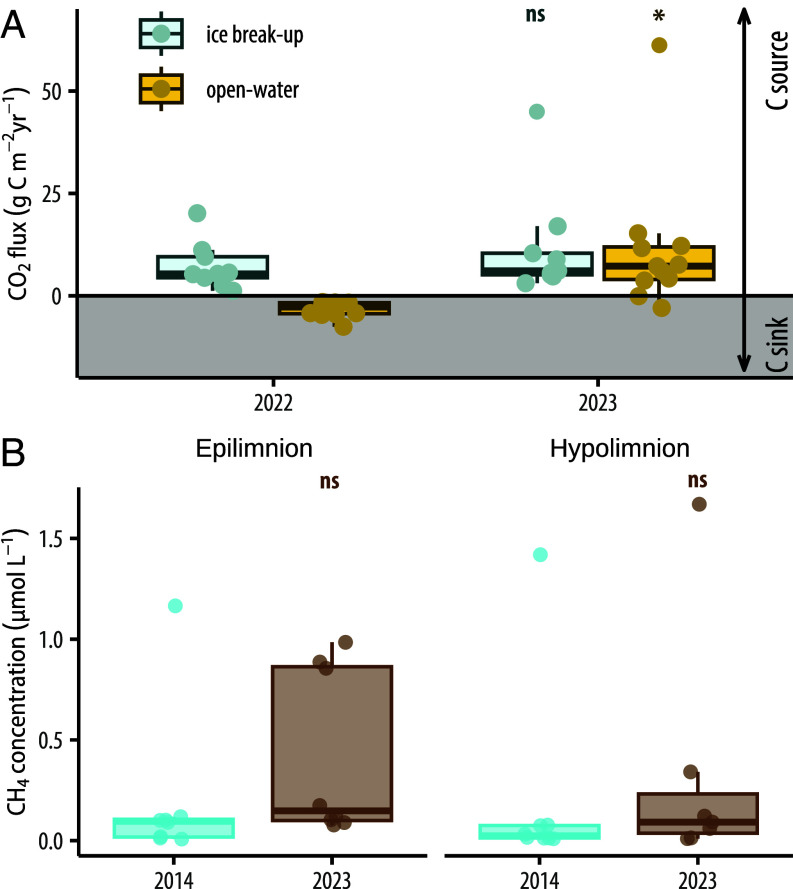
(*A*) Changes in CO_2_ flux at ice break-up (gray) in the spring and during summer (yellow) between 2022 and 2023 and (*B*) in epilimnetic and hypolimnetic CH4 concentrations measured in 2014 (blue) and 2023 (brown). Boxplot lines depict the median, outlines of the box are interquartile ranges. Each overlaid point represents a measurement from an individual lake.

CO_2_ emissions at lake ice-break-up were comparable before and after these large-scale hydrological shifts (32.9 to 36.3 mmol m^−2^ d^−1^; ~5.8 to 6.5 g C m^−2^ y^−1^, t = −1.12, df = 8, *P* = 0.29). However, CO_2_ fluxes during summer increased by 354% (t = −2.64, df = 10, *P* = 0.0248) and switched from uptake (−3.21 mmol m^−2^ d^−1^; −2.7 g C m^−2^ y^−1^) to efflux (6.87 mmol m^−2^ d^−1^; 7.2 g C m^−2^ y^−1^) (Fig. 7*A*), indicating that these lakes shifted from being carbon sinks to sources. Whole-lake methane concentrations were 72% higher in August 2023 than samples taken from the same lakes in August 2014 (0.37 ± 0.13 μmol L^−1^ vs. 0.22 ± 0.11 μmol L^−1^, respectively; Fig. 7*B*), although these differences were not significant (t = 2.14, df = 15, *P* = 0.06). In 2014, methane concentrations in epilimnetic and hypolimnetic waters were similar across lakes (0.23 ± 0.16 μmol L^−1^ vs. 0.23 ± 20 μmol L^−1^, respectively; t = 2.45, df = 6, *P* = 0.93). In 2023, the higher concentrations were driven primarily by increases in epilimnetic methane ranging from 34 to 1,039% higher than 2014 samples (Fig. 7*B*). Increased hydrologic connectivity, delivery of terrestrially derived compounds, and the lower water retention time of lakes are major drivers in organic matter decomposition for inland waters (50). Terrestrial inputs of C substrates in 2023 likely drove microbial processes leading to increased CO_2_ and CH_4_ (51).

Altered carbon cycling was further apparent in the elemental and isotopic signatures of particulate organic material (POM) in the water column across lakes. The C:N of POM increased 25% across lakes (*SI Appendix*, Fig. S15*A*), from 12.2 to 15.6 (Wilcoxon test *P* < 0.001), while mean δ^13^C of POM became less depleted by 25% (*SI Appendix*, Fig. S15*B*), from −28 to −21 (Wilcoxon test *P* < 0.001). Collectively, the depletion of N and shift to lighter δ^13^C in POM suggest stronger signatures from the DOM carbon pool in this material (52), as well as possibly the macrophyte carbon pool (*SI Appendix*, Fig. S15*C*), potentially reflecting high degradation rates of macrophytes with the abrupt loss of light penetration into these lake ecosystems.

## Discussion

**Fig. 8.**
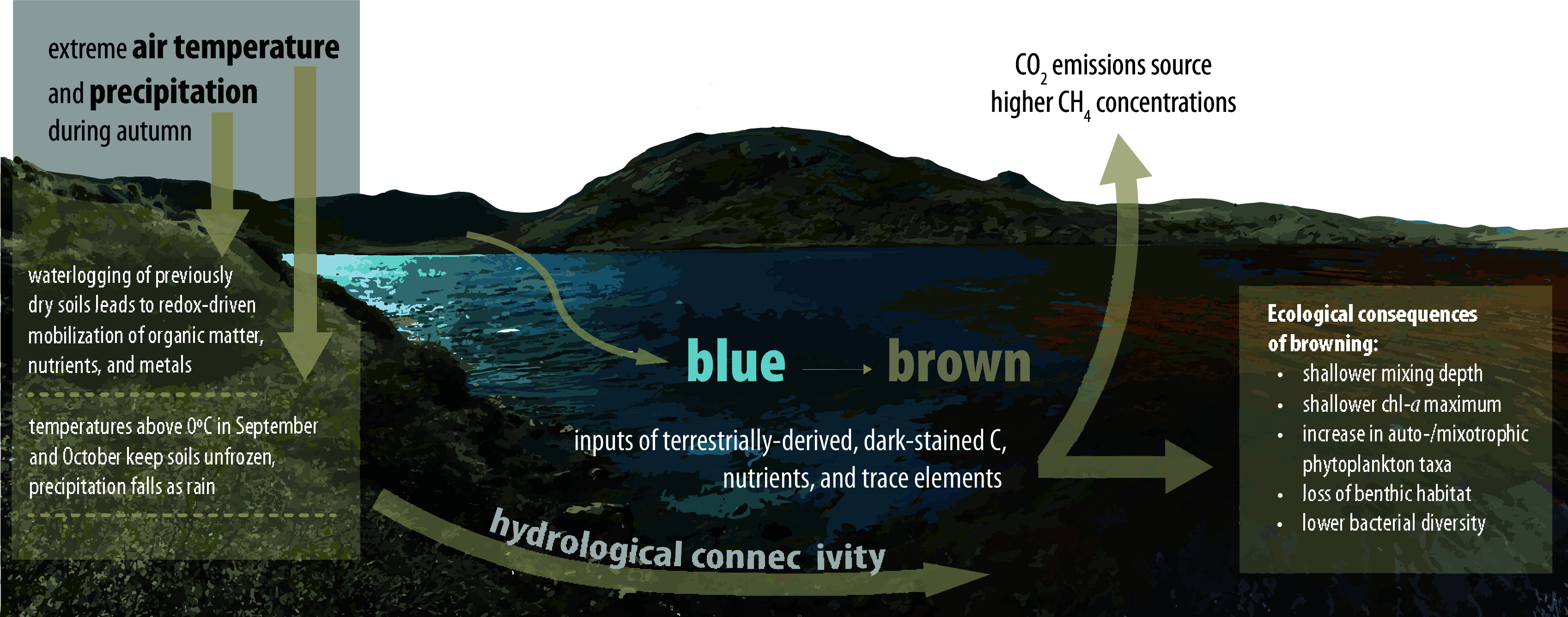
Summary of the effects of the compound climate extreme event of autumn 2022, indicating the impacts of the record heat and rainfall on terrestrial soil biogeochemistry, subsequent connectivity to lakes, and the ecological consequences to Arctic lakes.

The compound climate extreme events of autumn 2022 fundamentally and abruptly altered hydrologic connectivity and strengthened terrestrial–aquatic linkages, with myriad implications for the physical, chemical, and biological features of Arctic lake ecosystems (Fig. 8). Numerous lake characteristics were transformed between August 2022 and early July 2023, with darkening lake color leading to a reclassification of these lakes and imposing cascading effects on these systems. These darkly stained waters resulted from a phenomenal influx of terrestrially derived material, including DOC, that had been stored on the landscape with previously very limited hydrologic connectivity for at least decades, if not centuries (35, 36). An important outcome of the transformation of lakes from blue to brown is that the influx of allochthonous carbon led to an increase in CO_2_ flux from lakes to the atmosphere, switching lakes from summer sinks to summer sources of CO_2_; lake methane concentrations also increased. With more than 7,500 lakes in the affected area, this represents a regional shift in carbon dynamics. While compound climate extreme events are difficult to anticipate, their effects should be better explored and accounted for when quantifying and predicting how Arctic systems will change in the future.

The observed changes in numerous lake features were remarkably coherent, further highlighting the overwhelming strength of climate forcing of lake ecosystems by the record heat and rainfall in autumn 2022. A high degree of synchrony among lakes suggests the importance of large-scale factors in driving limnological responses. Past research has indicated that while climate variables reflecting energy inputs (i.e., air temperature, solar radiation, wind speed) tend to elicit strong coherence in lake ecosystem response across a region, those reflecting mass inputs (i.e., precipitation) often elicit more variable lake responses and hence a lower degree of synchrony (53). Furthermore, many biological responses exhibit low coherence across systems to climate forcing, likely due to the overriding effects of intrinsic ecological processes and lag times of responses (54). In contrast to prior work, we observed strongly coherent changes in numerous physical, chemical, and biological features across systems in response to a major hydrological change, underscoring the important and synergistic effect of the coupled extremes in both temperature and precipitation.

Changes in DOC quantity and quality indicated greater terrestrial inputs of this material after record heat and rainfall and are expected more broadly in the Arctic as the hydrological cycle intensifies (55). Strong responses were observed coherently across lakes in DOC quality, consistent with the conclusions of (56) who found that DOC quality parameters were more responsive to climate variability than DOC concentration. In west Greenland lakes, prior to the recently altered hydrology, DOC concentrations were already high but consisted of very low color material typical of semiarid regions (36), being predominantly autochthonous in origin (35). As a result, these lakes previously experienced limited landscape influence and served as storage sites of macrophyte- and algal-derived carbon. The transformation of lakes from blue to brown, owing to the rapid release of large quantities of terrestrially derived organic carbon previously stored in permafrost, drove a shift in the long-term summer status of these lakes as carbon sinks (35) to now being sources. While the increase in nitrogen-depleted biopolymers in 2023 suggested an influx of recalcitrant, likely heavily recycled DOM from soils, the simultaneous increase in lakewater nutrients, particularly nitrate concentrations, in early summer 2023 may have facilitated microbial degradation of this material (57) and contributed to the observed increase in CO_2_ flux.

The approximately 50% reduction of PAR in lake pelagic zones was likely a primary driver of the observed biological changes. Phytoplankton shifted from primarily autotrophic to mixotrophic groups, vertical positions of chlorophyll maxima rose in lake water columns, and changes in the balance of planktic to benthic diatom taxa suggested a loss of benthic habitats. Primary producers such as algae and macrophytes are typically more strongly affected by brownification than zooplankton, which are less dependent on light and therefore exhibit more complex responses to browning (22). Even with the changes in phytoplankton groups and distribution, integrated water column algal biomass did not change in pre- vs. postconditions, contrary to expectations based on remote sensing analyses (58), which suggest that increased hydrologic connectivity in Arctic lakes results in declining primary production. Future comparisons of whole lake metabolism in blue vs. brown states would provide a more comprehensive assessment of effects on lake production.

A major decline in prokaryotic diversity was also observed with the transformation of lake ecosystems. Other research (49) has also found a negative relationship between browning and taxon richness, with brownification inhibiting the growth and development of microbial communities. In these west Greenland lakes, dominant taxa shifted from the blue to brown lake states, with the picocyanobacterium *Cyanobium* becoming more widespread across brown lakes. *Cyanobium* is a common member of the phytoplankton of low to moderate productivity lakes (59); some strains appear to have an advantage at low irradiance levels (60). This taxon is capable of toxin production, suggesting the need for exploration of any links between toxin production and the browning of Arctic lakes.

Lake ecosystems of west Greenland are an example of systems at high risk for impacts of compound climate extreme events, owing to both the high prevalence and timing of these hazards, as well as environmental characteristics that increase the vulnerability of this area. West Greenland is one of many areas in the world situated in the path of ARs; importantly, ARs tend to strike this area in September (12), which is part of the autumn shoulder season (i.e., transition from summer thaw season) when air temperatures are typically dropping and biological activity in both terrestrial and aquatic systems is slowing, yet a relatively large proportion of the annual budget of DOC and other watershed-derived materials may be transferred to Arctic surface waters (61). Increasing autumn temperatures can shift the phase of precipitation during this period from snow to rain, increasing soil moisture conditions and landscape connectivity after senescence of terrestrial vegetation has occurred, amplifying the impacts of simultaneous extreme temperature and precipitation in autumn. The important impact of these coupled changes is evident in the fact that September 2003 experienced a similar magnitude of precipitation but milder warming (Fig. 1*B*), with no reported lake color change occurring (36). In addition to the prevalence and timing of these events, lake ecosystems in this area have heightened vulnerability to compound climate extremes, owing to the coupled long-term effects of low antecedent precipitation (average annual rainfall of 224 mm from 1975 to 2021, leading to long-term limited hydrologic connectivity) and soil conditions (specifically, the presence of continuous permafrost). Compound climate extreme events therefore have tremendous potential, as we observed, to trigger the release of large stores of organic carbon and other materials from the watershed into associated lake ecosystems. Considering the rapid intensification of the Arctic hydrological cycle (62) and the emerging new Arctic climate having warmer, wetter autumns dominated by a rain precipitation phase (63, 64), the effects that we observed in west Greenland from record heat and rainfall in autumn 2022 may portend the types of lake ecosystem changes that can be anticipated more broadly in the Arctic, and underscore the need to better understand the ecological effects of changing climate in the shoulder seasons.

Ecosystem transformations persist over different time scales but can have tremendous ecological and human impacts with potentially long-lasting effects. Perhaps one of the best examples of the destructive and highly disruptive nature of ecosystem transformation driven by a series of ARs is the winter of 1861-62 megaflood in California, which struck after decades of drought (65). Large regions of the state were flooded and transformed into brown, inland seas for months. While this ecosystem transformation eventually reverted, the economic impacts were staggering, with 25% of the state’s economy destroyed and the state of California declaring bankruptcy as a result. The transformation of west Greenland lake ecosystems driven by a series of ARs has persisted at least through the summer of 2024, and it remains to be seen if and when they might revert back to a blue state. Our results reveal that, while in their darker, brown state, these lakes are a larger source of GHGs, and tens of thousands of lakes in west Greenland were affected by these compound climate extreme events. The brown state also has implications for Greenlandic communities, as some settlements use lakes for drinking water. Elevated DOC and metal concentrations can reduce drinking water quality through taste and odor problems and pose several health risks, including disinfection by-products, microbial exposure, and metal toxicity (66, 67). These risks can be mitigated with the implementation of new infrastructure; communities will have to consider costs of remediation vs the frequency and severity of these threats. Lake ecosystems of west Greenland coherently crossed a tipping point owing to compound climate extremes, underscoring the value of better integrating the potential for such surprises into future scenarios and adaptation plans. This supports recent calls (68) to better account for high-impact extreme events in climate risk assessments and plausible scenario planning in an effort to better anticipate and adapt to future conditions.

## Materials and Methods

### Experimental Design.

For 10 lakes around Kangerlussuaq, Greenland, we compared July and/or August 2023 (“post”) values to corresponding data prior to September 2022 (“pre”) using paired *t* tests. For post data, July 2023 samples were collected between June 30-July 10, and August 2023 between August 12 to 18. The pre data primarily span from 2013 to 2022. From 2013 to 2019, lakes were sampled in early July annually. The COVID-19 pandemic resulted in no observations in summer 2020, and only late summer (early to mid-August) observations on a subset of lakes in 2021 and 2022. Data were also collected from a subset of lakes under ice cover in April 2022 and 2023. To demonstrate persistence of the postevent effects, we also include data from July 2024 in time series datasets when available. Lakes in this area are covered by ice from October/November to May/June. The sampled lakes are fishless, and range in maximum depth from 11 to 30 m. The area is underlain by continuous permafrost. Average annual rainfall is 224 mm (spanning 1975 to 2021), resulting in very limited surface hydrologic connectivity of these lakes with the landscape (35, 36). These lakes typically have high concentrations of low color DOC, typical of lakes situated in semiarid landscapes (36). These lakes are part of the 25% of total circumpolar lake area situated in arid, low-relief regions (27).

### Lake Sampling.

All lake sampling was conducted from a rubber raft in the middle of the lake. Water-column PAR was quantified using a BIC submersible profiling radiometer coupled with a deck radiometer (Biospherical Instruments, San Diego, CA). Vertical temperature profiles were collected with a multiprobe during July and August (HydroLab until 2015, YSI EXO from 2016, YSI EXO and Turner C3 in 2019) from 2013 to 2023 and daily averaged high-frequency temperature time-series collected by thermistors during July and August 2014 to 2023 (Onset Hobo Pendant).

Water samples were collected for a suite of analyses, at the time points indicated throughout the text and figures, and using techniques described in *SI Appendix*. CO_2_ concentration was measured with a handheld Vaisala GMP252 in July-August 2022 and 2023, as well as under ice in April 2022 and 2023. Vertical tows for zooplankton were collected in August 2014 and 2023 with a 60-μm mesh net. Tows were collected over the majority of the water column, integrating the same volume in both years. Samples from both years were preserved in 70% ethanol before identification.

Lake sediment cores were collected from the deep area of each lake using a Pylonex HTH gravity corer. Sediments were extruded in the field in 0.25 cm increments, the finest resolution possible. Based on prior research of these lakes, the top 0.25 cm of sediment cores captures the previous 6 to 12 mo of sediment accumulation (69).

### Analytical Methods.

The depth of 1% PAR attenuation was calculated from PAR profiles. Thermal structure metrics (Schmidt stability and mixing depth) were calculated using the function *schmidt.stability* and *thermo.depth* where the depth of mixing is defined as the depth of maximum density gradient (package *rLakeAnalyzer*).

Water chemistry was analyzed according to standard methods unless otherwise noted and is described in *SI Appendix*.

DNA extractions of 2019 and 2023 samples took place at the University of Maine eDNA CORE Lab using DNeasy DNA extraction kits following the manufacturer’s protocol. All samples were eluted to 100 μL and sequenced on an Illumina MiSeq platform at the University of Maine eDNA Services Center using primers targeting the 16S V4 gene region (*SI Appendix*, Table S4). Six lakes were used to compare changes pre- and post-2023. Additional details of eDNA analysis are provided in *SI Appendix*.

Headspace concentrations of methane were determined from scintillation vials; methane values from the GC were converted to μmol L^−1^ by correcting for water and headspace volume using the molar volume of methane gas at 20 °C and a Bunsen coefficient of 0.035 (70). The open-water CO_2_ flux from each lake was estimated from CO_2_ concentration data. Additional details are provided in *SI Appendix*.

Zooplankton from both years were counted and identified to the lowest taxonomic level under 80× magnification. Zooplankton biomass was estimated by multiplying the density of each zooplankton genus by the average individual weight of each genus from various peer-reviewed literature (71).

The upper 0.25 cm of sediments collected in 2023 were analyzed for diatom taxa and compared to those from surface sediments from the same lakes collected in 2013 (6). Changes in the percent relative abundances of *D. stelligera*, an indicator taxon of shallower lake mixing depths (48), and the P:B of taxa were determined to assess lake habitat changes with the transformation to brown lakes.

### Statistical Analysis.

To assess differences in lake characteristics between 2023 and years before the extreme climate event, we used a paired *t* test. In the cases of C:N and δ^13^C, a Wilcoxon test was used as data characterizing the earlier period were not resolved to the lake level (52), and the homogeneity assumption was not met. To demonstrate whether effects were sustained in 2024, any data available from July 2024 were also compared to pre-event values using paired *t* tests.

## Supplementary Material

Appendix 01 (PDF)

Movie S1. Animation of total column precipitable water standardized anomalies for September 2022 using 3-hourly time slices from reanalysis. Anomalies in reference to 1951–2000 climatology. Dataset: ECMWF Reanalysis Version 5 (ERA5) (*15*), processed using ClimateReanalyzer.org.

Movie S2. Animation of total column precipitable water standardized anomalies for October 2022 using 3-hourly time slices from reanalysis. Anomalies in reference to 1951–2000 climatology. Dataset: ECMWF Reanalysis Version 5 (ERA5) (*15*), processed using ClimateReanalyzer.org.

Movie S3. Animation of total column precipitable water standardized anomalies for July 2023 using 3-hourly time slices from reanalysis. Anomalies in reference to 1951–2000 climatology. Dataset: ECMWF Reanalysis Version 5 (ERA5) (*15*), processed using ClimateReanalyzer.org.

## Data Availability

Data have been contributed to the NSF Arctic Data Center (10.18739/A2TD9N97F) (46). All other data are included in the article and/or supporting information.
