# Supplementary material for: Abrupt transformation of west Greenland lakes following compound climate extremes associated with atmospheric rivers
Source: Proc Natl Acad Sci U S A. 2025 Jan 21;122(4):e2413855122. doi: 10.1073/pnas.2413855122 (PMC11789078; doi:10.1073/pnas.2413855122)
Supplement: Supplementary file 1 — Appendix 01 (PDF) [file pnas.2413855122.sapp.pdf]

PNAS

Supporting Information for

Abrupt transformation of West Greenland lakes following compound climate extremes associated with atmospheric rivers

Jasmine E. Saros,[1,*] Václava Hazuková,[1] Robert M. Northington,[2] Grayson P. Huston,[1] Avery Lamb,[1] Sean Birkel,[3] Ryan Pereira,[4] Guillaume Bourdin,[5] Binbin Jiang,[6,7] Suzanne McGowan[8]

[*]Corresponding author: Jasmine E. Saros
Email: jasmine.saros@maine.edu

This PDF file includes:

Supporting text
Figures S1 to S15
Tables S1 to S4
Legends for Movies S1 to S3
SI References

Other supporting materials for this manuscript include the following:

Movies S1 to S3

Supporting Information text
Methods

Additional information on water sampling and analysis
Using bottles rinsed with deionized (DI) water, samples for ANC and water isotopes were collected by submerging bottles under the lake surface. All other water samples were collected with a non-metallic van Dorn sampler from three depths: the surface mixed layer (epilimnion), the deep layer (hypolimnion), and the transition zone between them (metalimnion). For all nutrients, DOC, color, and POM (for C:N seston ratios and $\delta^{13}C$), samples were collected into HCl-washed bottles; samples for dissolved nitrogen, bulk DOC parameters, and color were filtered with Whatman GF/F 0.7 μm filters, for silica were filtered with 0.4 μm polypropylene filters, and for POM were collected onto Whatman GF/F 0.7 μm filters. For chlorophyll *a* and photosynthetic pigments, samples from each depth were collected into dark, DI-rinsed bottles, filtered onto Whatman GF/F 0.7 μm filters, and frozen until analysis. For total metals, samples were transferred directly from the van Dorn sampler into nitric-acid washed bottles. For methane in 2014, 10mL of water from each layer were injected into pre-evacuated, He-filled, 20mL scintillation vials acidified with 0.1 mL of 0.1N HCl, while in 2023, 10mL of water from each layer was injected into a pre-evacuated N_2-filled 12-ml vial filled with 0.1mL of 0.1N HCl. Water samples for environmental DNA (eDNA) analysis were collected from the epilimnion, with samples transferred directly from the van Dorn sampler into sterilized bottles before a subset (250 mL) was filtered onto 0.8 μm Isopore polycarbonate filters and frozen until further analysis.

A Varian Cary-50 Ultraviolet-Visible spectrophotometer (Agilent Technologies) was used for all spectrophotometric analyses. ANC was determined by titration. Water isotopes were analyzed with a Picarro L2130i laser cavity ring-down spectrometer. Results are reported relative to SMOW (Standard Mean Ocean Water); precision is 0.1‰ for $\delta^{18}O$ and 0.1‰ for δD. Ammonium and nitrate were analyzed with flow injection analysis using the phenate and cadmium reduction methods, respectively, on a Lachat Quikchem 85 (Hach Company). TP was determined from whole-water samples using persulfate digestion followed by the ascorbic acid method, measured on a spectrophotometer. Dissolved silica was measured with the heteropoly blue method with a spectrophotometer. True color was based on absorbance at 457.5 nm measured with a spectrophotometer. Chlorophyll *a* samples were extracted in 90% acetone, clarified via centrifugation, and concentrations were determined with a spectrophotometer. Analyses were performed within 3 weeks of filtration according to standard methods. Photosynthetic pigment analysis was based upon standard high-performance liquid chromatographic (HPLC) separation of chlorophylls, chlorophyll degradation products, and carotenoids using a mixture of 80:15:5 acetone: methanol: water. An HPLC equipped with a photodiode array detector was used to separate, identify, and quantify photosynthetic pigments by comparison of spectra to known standards (*1*; DHI, Denmark).

DOC concentration was determined with a Shimadzu TOC-5000 total organic carbon (TOC) analyzer (Shimadzu Corporation) by high-temperature catalytic oxidation. Spectral absorbance was measured with a spectrophotometer and absorption coefficients obtained with the equation:

$$a(\lambda) = \frac{2.303A(\lambda)}{L} \quad (1)$$

In this equation, *a* represents the Napierian absorption coefficient (m^{-1}) at wavelength λ, *A* is absorbance value at wavelength λ, and *L* is the cuvette path length, 0.01 m. $SUVA_{254}$ and a^*_{375} were determined from absorbance values at 254 and 375 nm, respectively, and divided by DOC concentration. Spectral slopes were measured from 275 to 295 nm and from 350 to 400 nm; the ratio of these slopes was calculated to produce S_R (*2*).

DOM composition was determined using an LC-OCD-OND that allows ~1 ml of unfiltered water to be injected onto a size exclusion column (SEC; 2 ml min^{-1}; HW50S, Tosoh Bioscience) with a phosphate buffer (potassium dihydrogen phosphate 1.2 g L^{-1} plus 2 g L^{-1} di-sodium hydrogen phosphate x 2 H_2O, pH 6.58) and separated into five "compound-group specific" DOM fractions. The resulting compound groups are identified using unique detectors for organic carbon, UV-amenable carbon and nitrogen (*3*). These compound groups include biopolymers (BP; high molecular weight polysaccharides and proteins), humic

substances (HS), building blocks (BB; lower molecular weight HS), low molecular weight acids (LMWA), and low molecular weight neutrals (LMWN; amphiphilic/neutral compounds including alcohols, aldehydes, ketones, and amino acids) (*4*). All peaks were identified and quantified with bespoke software normalized to International Humic Substances Society humic and fulvic acid standards, potassium hydrogen phthalate and potassium nitrate. LC-OCD-OND can be further used to determine the nominal molecular weight of HS (Mn) in natural waters relative to the known molecular weight of IHSS Humic Acid and Fulvic Acid and is able to quantify organically bound nitrogen in the humic substances and biopolymer compound groups, in addition to inorganically bound NH_4^+ and NO_3^-, by passing the sample through a UV-glass capillary reactor that converts nitrogen species to NO_3.

Water samples for metal analyses were acidified to 1% nitric acid using Optima nitric acid and analyzed using a Thermo Element XR ICP-MS (Thermo Scientific) with a ESI Apex2Q sample introduction system with a ESI PFA-ST nebulizer and 100uL/minute sample uptake. The instrument was tuned and calibrated before each run. An external standard (SLRS-6 from National Research Council Canada) was analyzed with each run.

POM analysis for $\delta^{13}C$ and $\delta^{15}N$ isotopes was completed using an elemental analyzer (EA, Flash2000) coupled to an isotope ratio mass spectrometer (IRMS) (Thermo Scientific, USA). $\delta^{13}C$ and $\delta^{15}N$ values are reported relative to standards VPDB (Vienna Pee Dee Belemnite) and Air, respectively, calculated with calibration equations from (*5*). Carbon and nitrogen content of POM samples were calculated using the thermal conductivity detector trace of the EA using a linear standard curve of different amounts of sulfanilamide (41.84% C, 16.27% N, Thermo Scientific), nicotinamide (59.01% C, 22.94% N, Thermo Scientific) and L-aspartic acid (36.09% C, 10.52% N, Thermo Scientific).

<u>Environmental DNA analysis</u>

eDNA samples were first amplified using primers with tails for Nextera indexing and were run in 25 μl reactions using previously published PCR reaction mixtures and thermocycler conditions (*6*). Amplicons were visualized on an agarose gel and target sizes were selected and cleaned with a Zymo Select-a-Size Clean & Concentrator MagBead Kit following manufacturer's instructions. Cleaned PCR products underwent a second PCR to add Illumina indices to each sample before indexed amplicons were paired and sequenced on an Illumina MiSeq using a v3 reagent kit. Prior to bioinformatic analysis, relevant sequences from 2015 were downloaded from the European Nucleotide Archive and split into individual forward and reverse reads using the SRA-Toolkit. Bioinformatic analysis was then performed on raw sequences from 2015, 2019, and 2023 using the DADA2 package in R Studio (version 4.2.1) following the Ishaq DADA2 bioinformatics pipeline (*7*; Ishaq, AVS 554 pipeline). Based on sequence quality, only forward sequence reads were used for analysis, with 19 samples containing 1,493,909 raw reads and 1,444,696 filtered reads consisting of 2,951 sequence variants (SVs). 293 bimeras were identified and removed, and the remaining 2,658 SVs were assigned taxonomy using the Silva Prokaryotic Database (version 138.1). SVs were then aggregated by genus level, leaving a total of 217 individual taxa. A total of 3 negative controls containing 23 taxa were then investigated and removed from the dataset, and all samples containing the same sequences as the negative controls were removed from the dataset, leaving a total of 194 taxa.

To assess eDNA changes, the package phyloseq (*8*) was used to analyze all filtered sequences. Bacterial richness was estimated using Hill's diversity indices which measure absolute species richness (q=0), the exponential of Shannon diversity (q=1), and inverse Simpson (q=2). A t-test was then performed on q=2 values to compare alpha-diversity of the more abundant species between sample periods (i.e., pre and post 2023). Beta-diversity analysis was conducted based on Jaccard distance matrices to calculate species similarity and overlap between sample periods. A PERMANOVA analysis was then performed to test for significant changes in community composition between sample periods. To account for different filter sizes used in 2015 (0.2 μm) compared to 2019 and 2023 (0.8μm), separate Hills diversity indices, t-test, and PERMANOVA analyses were performed to assess differences between sample years with similar methodologies. The three overlapping lakes displayed trends similar to those observed with the entire dataset, though changes in alpha diversity (t=1.275, df=2.8976, p=0.2924) and beta diversity (F=2.4514, R2=-0.37998, p=0.100) were not statistically significant.

<u>Greenhouse gases</u>

In 2014, headspace concentrations of methane were determined from scintillation vials, acidified with 0.1mL of 0.1M HCl per 10mL of water, using a Shimadzu GC8A (Shimadzu Corporation) with flame ionization detector with a 1/8 inch x 1-m molecular sieve 5A column and ultrahigh purity N_2 carrier gas. Precision of analysis was 10 μL L^{-1} = 0.9%, with a detection limit of 0.2 μL L^{-1}. In 2023, headspace concentrations (similarly acidified as above) were determined using GC2 Trace1600 TCD (Methanizer-) FID Thermo Scientific Trace1600 GC with TCD and (methanizer-)FID detector (Interscience, Breda) using split/splitless injection using high quality He or N2 gas. For both years, methane values from the GC were converted to µmol L^{-1} by correcting for water and headspace volume using the molar volume of methane gas at 20°C and a Bunsen coefficient of 0.035 (9).

The open-water CO_2 flux from each lake was estimated from CO_2 concentration data measured by handheld Vaisala GMP252 in July-August in 2022 and 2023 following Fick's law of diffusion,

$$F_{CO_2} = k_t * ((pCO_{2_{water}} * K_H) - pCO_{2_{air}}) \quad (2)$$

where k_t is the gas transfer velocity at time t, a measure of turbulence of the water, and pCO_2 are the partial pressures of CO_2 in water and in the air. Partial pressure of CO_2 in water is multiplied by Henry's constant for CO_2 at a given temperature and salinity (*10*). For air pCO_2, we used the average monthly CO_2 measurements from the closest ICOS station on Disko Island, West Greenland operated by the University of Copenhagen, Denmark. Gas transfer velocity of CO_2 varies with temperature of water and was calculated as

$$k_t = k_{600} \left(\frac{Sc_t}{Sc_{600}}\right)^n \quad (3)$$

where Sc_t is the Schmidt number at a particular temperature calculated following (*10*), n was to -0.5 (*11*). k_{600} is a gas transfer velocity standardized for a Schmidt number of 600; here we calculated k_{600} using a common wind-based model (*12*):

$$k_{600} = 2.07 + \left(0.215 * {U_{10}}^{1.7}\right) \quad (4)$$

where U_{10} is the wind speed at the height of 10 m. k_{600} is a source of major uncertainty in flux calculations and while wind speed is a major driver of turbulence, other factors like lake size play a role (*13*). The CO_2 evasion at ice-melt was estimated using measurements taken with Vaisala GMP252 under ice in April 2022 and 2023. In April 2023, 6 autonomous, *in-situ* IRGA sensors Pro-Oceanus MiniCO_2 were deployed, measuring aquatic CO_2 concentration every 6 hours from the beginning of April until the beginning of July. These probes indicate that CO_2 accumulates under ice until ~2 weeks before ice-out when moats begin to form and there is an established connection between parts of the lake surface and the atmosphere. Ice break-up flux estimates from continuous data correspond well to the estimates based on Vaisala GMP252 measurements (mean difference of 0.3+-1.6 g C m^{-2} yr^{-1}). Thus only comparisons based on Vaisala GMP252 measurements available for both years, before and after the described hydrological transformation, and from more lakes, are shown.

Satellite data

Atmospherically corrected Sentinel 2a and 2b MSI level 2A data were downloaded from the Copernicus Data Space, covering an area of approximately 300 km^2 in West Greenland from 65°39'55.8"N to 68°30'45.0"N and from 54°49'12.4"W to 48°18'59.4"W (tile#: T21WXP, T21WXQ, T21WXR, T22WDA, T22WDU, T22WDV, T22WEA, T22WEU, T22WEV). The scene classification map (SCL) of each satellite image was used to retain only data for pixels classified as vegetated (flag #4) and non-vegetated (flag #5). Average summer NDVI were computed at 20 m spatial resolution over July and August of each year from 2019 to 2023, to identify anomalies in vegetation density as a proxy for increased rainfall and run-off in the region. NDVI was computed as the ratio of the difference by the sum of MSI bands B08 and B04 ((B08 - B04) / (B08 + B04)). All NDVI scenes from each tile number were binned, taking the median of all available data for a given band over a one-month period. These monthly binned products were resampled at 300 m spatial resolution and merged onto an equally spaced 300 m spatial resolution latitude and longitude grid covering the entire region. The NDVI composites of July and August were then averaged to represent the

average growing season's NDVI for each year. The NDVI anomaly was computed as the difference between the average NDVI in summer 2023 and the average NDVI of multiple summers (2019, 2020, 2021, 2022) prior to the initiation of AR activity in September 2022.

All lakes in each MSI Level 2A scene were first detected using the water flag of the SCL product (flag #6) distributed with Level 2A products. A significant number of pixels impacted by topographic shadows were mis-classified as water in the SCL product. Therefore, these false positives were filtered out based on a near-infrared false color threshold (i.e. when $20 \times B8A^2 \times 20 \times B04 / max(20 \times B8A^2 \times 20 \times B04) > 0.1$). A single mask of lakes located in each tile area was created based on all scenes of a given tile number from June to September of each year (2019 to 2023). Only pixels identified at least twice as water were used to create an initial lake mask. Properties of each lake patch were extracted using the matlab function "regionprops" (i.e. Centroid, Area, MinorAxisLength, MajorAxisLength, Perimeter, Circularity, Orientation, and Solidity). We only retained lake patches that did not overlap with the domain border, were larger than 25 pixels and smaller than 10 million pixels in area, had a minor axis length greater than 5 pixels, and were completely clear of cloud, cirrus, and cloud shadow. Small patches (< 300 pixels) with solidity < 0.3 were also filtered out to minimize topographic shadow artifacts. The deepest points of lakes were computed as their Chebyshev center which are defined as the center of the largest circle fitting in each lake polygon (*14*). Because Sentinel 2a and 2b tiles overlap in space at the border of tiles, a single lake can be detected in multiple tiles. Duplicate lakes were avoided by removing lakes patches from the list when the closest lake was below a distance threshold defined as the knee point of the relation between the distance to the closest lake and its associated number of lake duplicates detected.

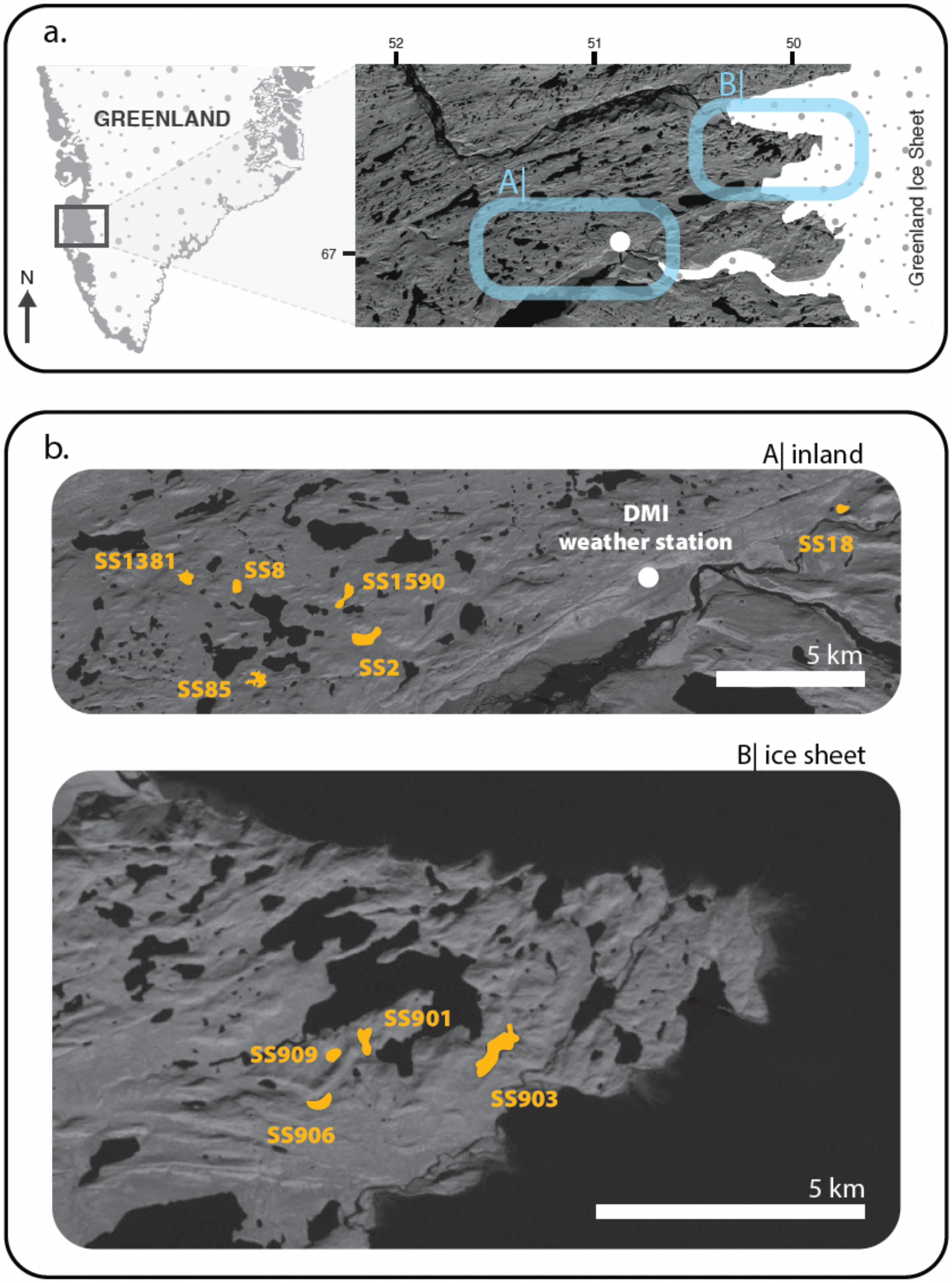

Fig. S1. The study sites are in West Greenland; the inset in part a. shows the location of two areas where all studied lakes are located along the gradient between Kellyville (A) and the Greenland Ice Sheet (B). Panel b shows the exact location of each lake in the two areas; lake names in orange text. White point shows the location of the Danish Meteorological Institute (DMI) weather station.

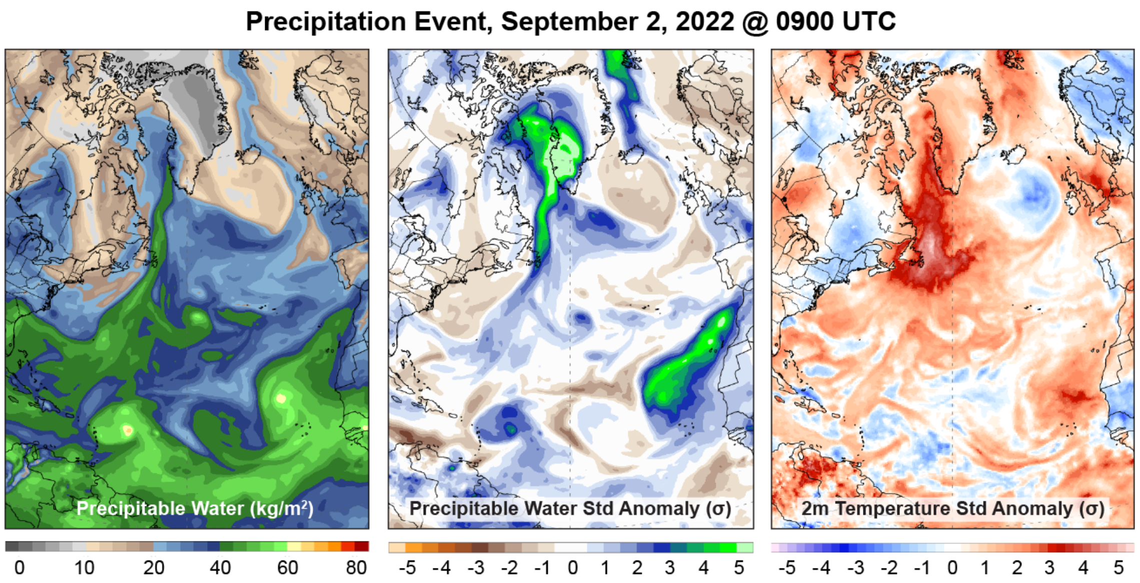

Fig. S2. Maps showing total column precipitable water (left) and standardized anomalies of precipitable water (center) and 2-meter air temperature (right) on September 2, 2022 during a major atmospheric river event. Anomalies in reference to 1951–2000 climatology. Dataset: ECMWF Reanalysis Version 5 (ERA5) (*15*), processed using ClimateReanalyzer.org.

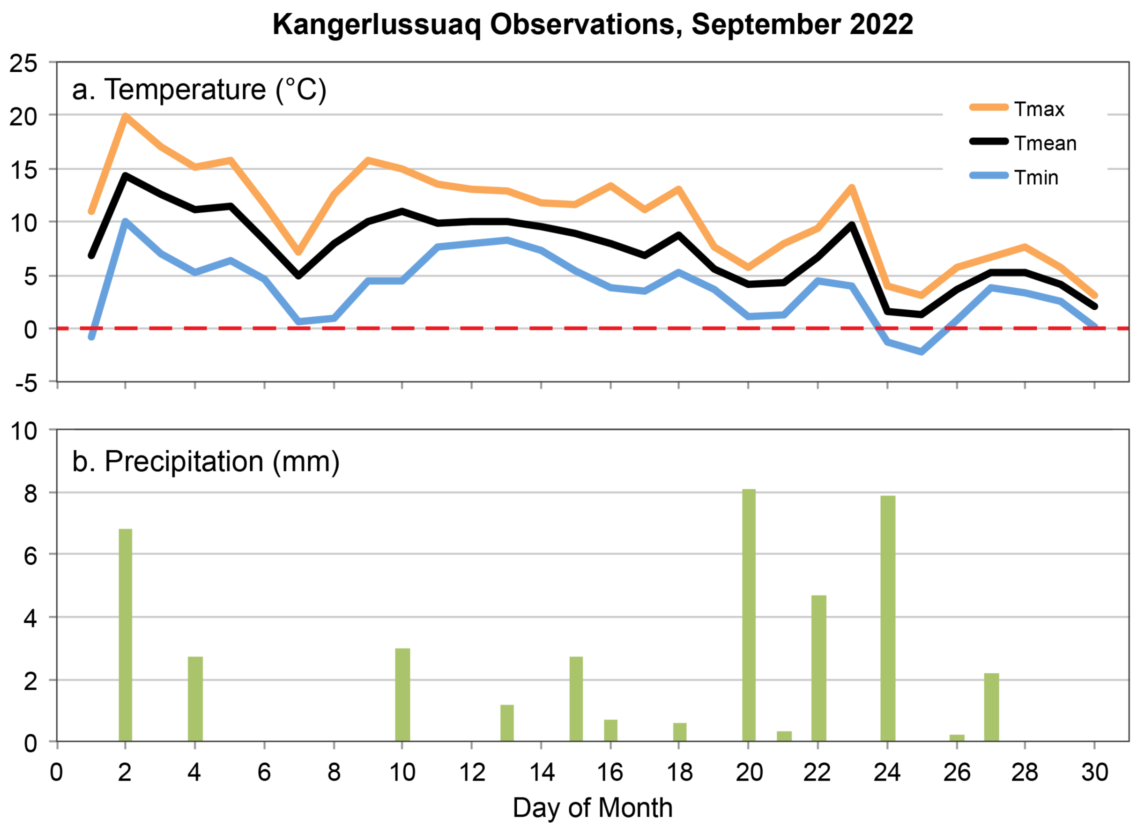

Fig. S3. (a) September 2022 daily maximum, mean, and minimum temperature observations from a weather station at Kangerlussuaq. (b) Precipitation observations for the same time period. Data downloaded from dmi.dk.

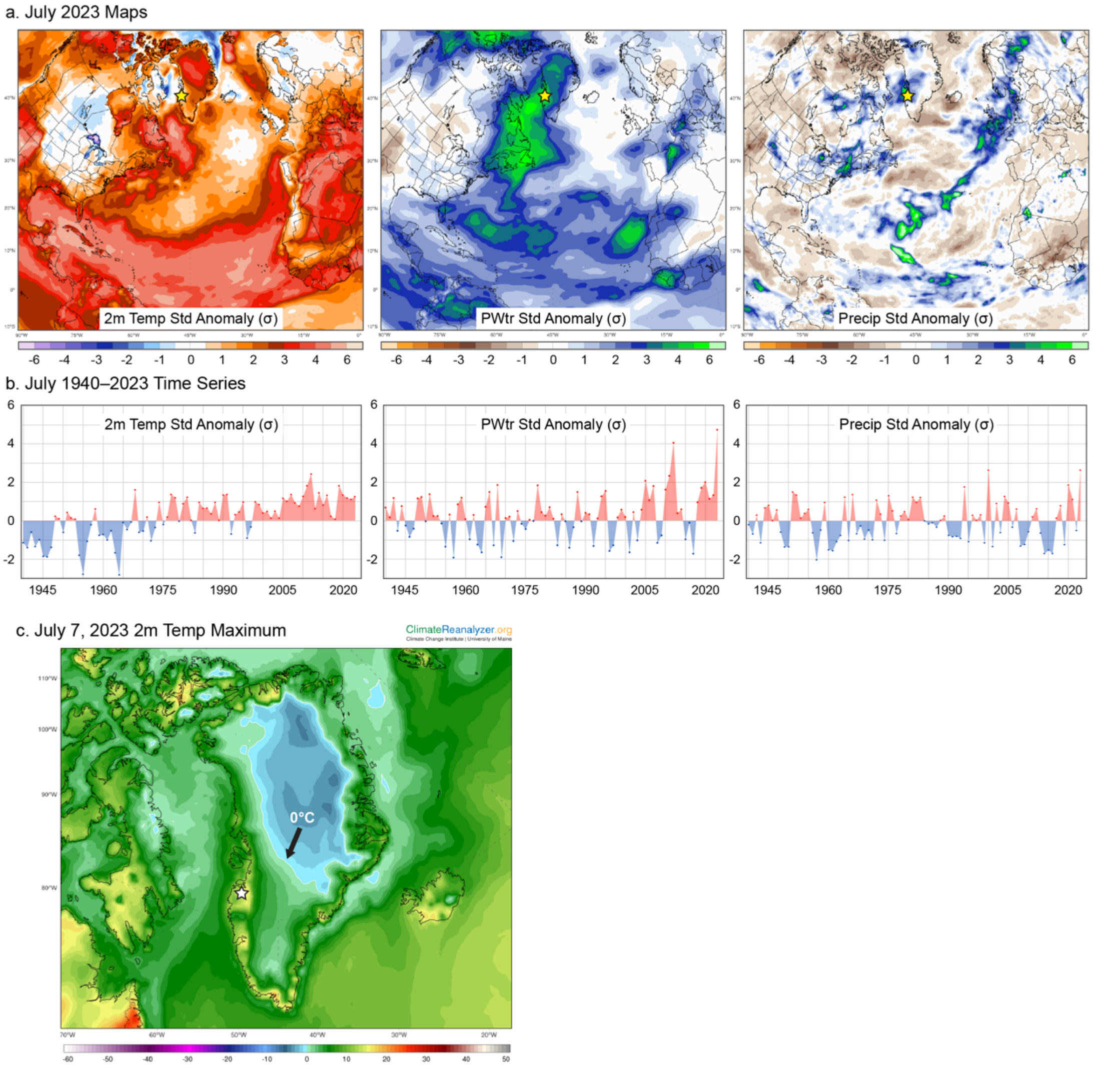

Fig. S4. (a) Maps showing standardized anomalies of 2-meter air temperature (2m temp), precipitable water (pwtr), and precipitation (precip) for July 2023. (b) Time series of standardized anomalies for 2m temp, pwtr and precip for July 1940–2023. (c) Map of the maximum 2m Temp for July 7, 2023 showing a warm event in which temperatures reached above freezing across almost half of the Greenland ice sheet. The study location is marked with a star. Anomalies in reference to 1951–2000 climatology. Dataset: ECMWF Reanalysis Version 5 (ERA5) (*15*), processed using ClimateReanalyzer.org.

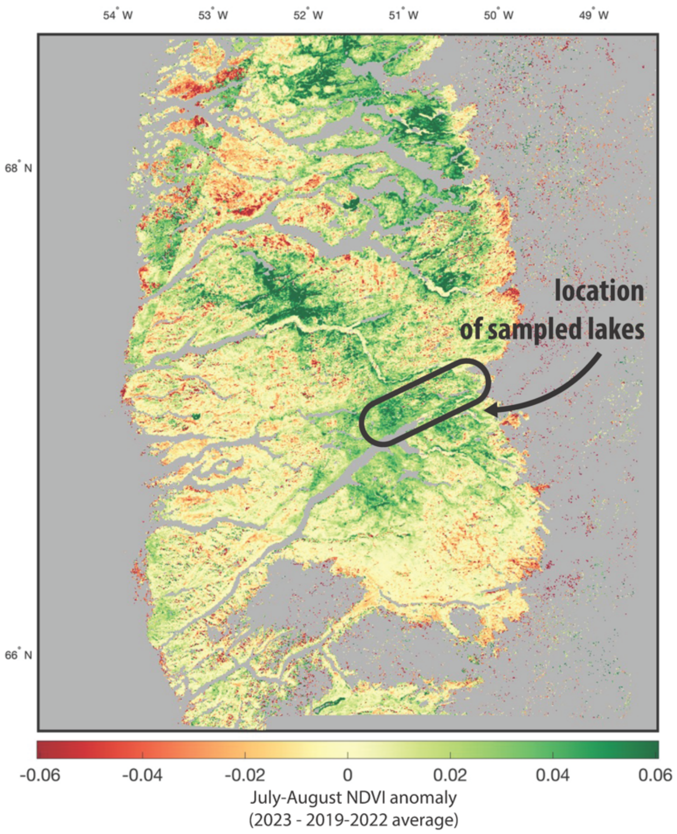

Fig. S5. Normalized Difference Vegetation Index (NDVI) anomaly in summer 2023 relative to pre-September 2022 (from 2019 to 2022), estimated from Sentinel 2a&b MSI composites at 300m spatial resolution. Lake study region indicated with black oval.

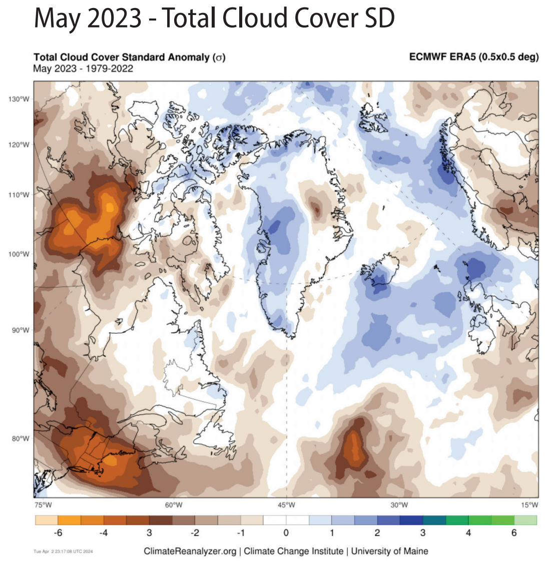

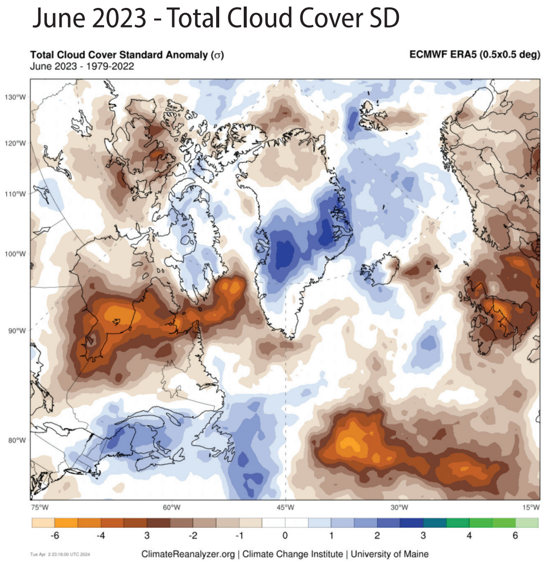

Fig. S6. Total cloud cover standard anomaly in May 2023 (left) and June 2023 (right) compared to average from 1979-2022. Maps were generated in Climate Reanalyzer using ERA5 data.

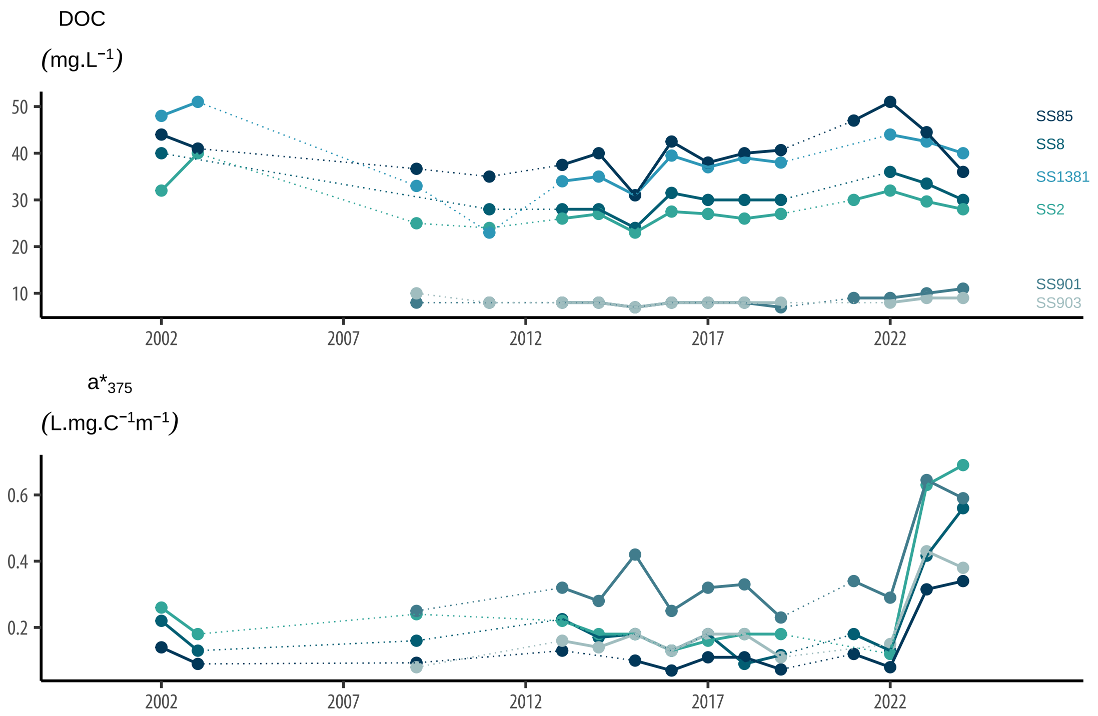

Fig. S7. Time-series of summer DOC concentrations (top) and a^*_{375} (bottom) since 2002 for six lakes. Lake names are indicated to the right in the top panel. Data before 2013 were reported in (*16*).

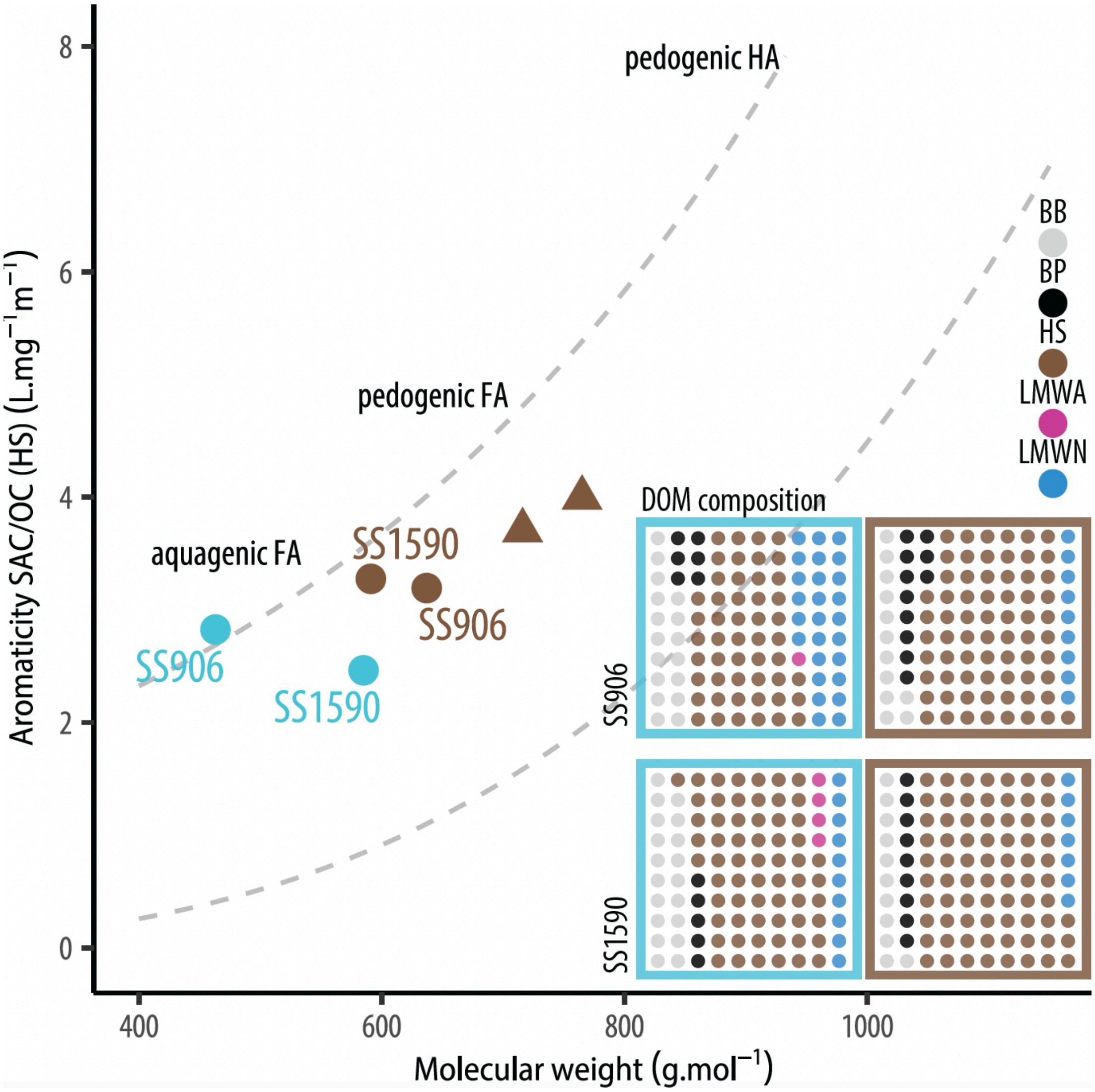

Fig. S8. Humification pathway plot of molecularity and $SUVA_{254}$ of the humic substances of DOM. Blue circles show the position of lakewater DOM samples in 2018 and brown circles in 2023. Brown triangles represent DOM watershed samples in 2023. The inset plot shows how DOM composition in samples from the same lakes changes from 2018 to 2023. Grey - building blocks, black - biopolymers, brown - humic substances, pink - low molecular weight acids, blue - low molecular weight neutrals.

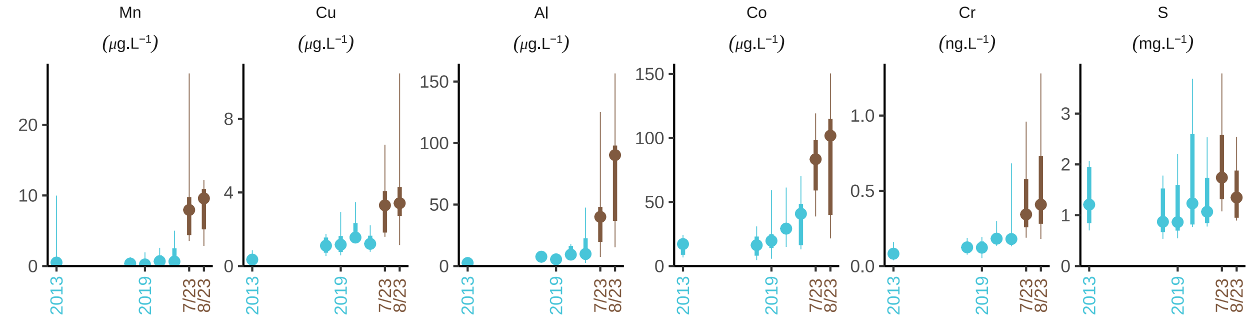

Fig. S9. Epilimnetic concentrations of manganese, copper, aluminum, cobalt, chromium, and sulfur. July and August 2023 (brown) data are compared to summer data (June - August) collected since 2013 (blue). Central point is the median value across lakes, the thicker line depicts the interquartile range, and the thinner line shows values within the 90th percentile.

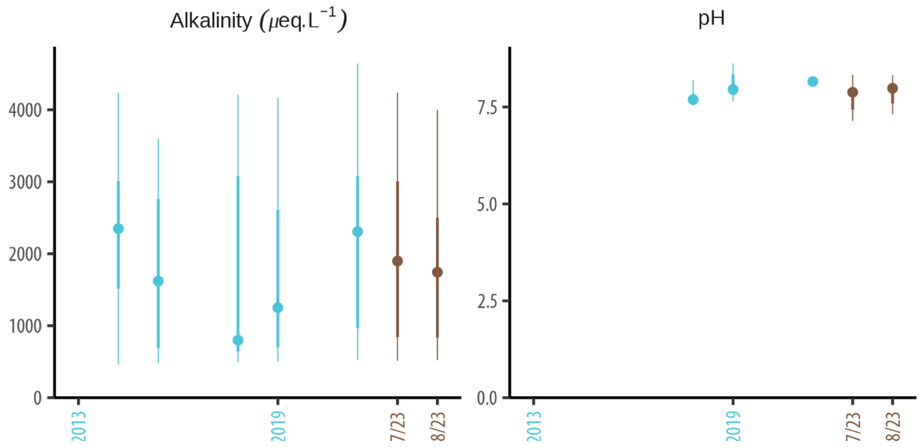

Fig. S10. Epilimnetic alkalinity and pH. July and August 2023 (brown) data are compared to summer data (June - August) collected since 2014 (alkalinity) or 2018 (pH) (blue). Central point is the median value across lakes, the thicker line depicts the interquartile range, and the thinner line shows data within the 90th percentile.

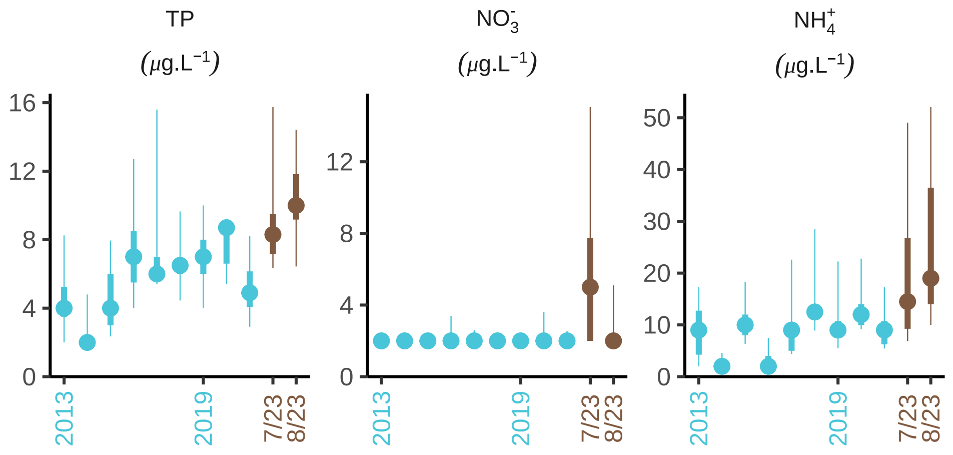

Fig. S11. Epilimnetic concentrations of total phosphorus (TP), nitrate (NO_3^-) and ammonium (NH_4^+). July and August 2023 (brown) data are compared to summer data (June - August) collected since 2013 (blue). Central point is the median value across lakes, the thicker line depicts the interquartile range, and the thinner line shows data within the 90th percentile.

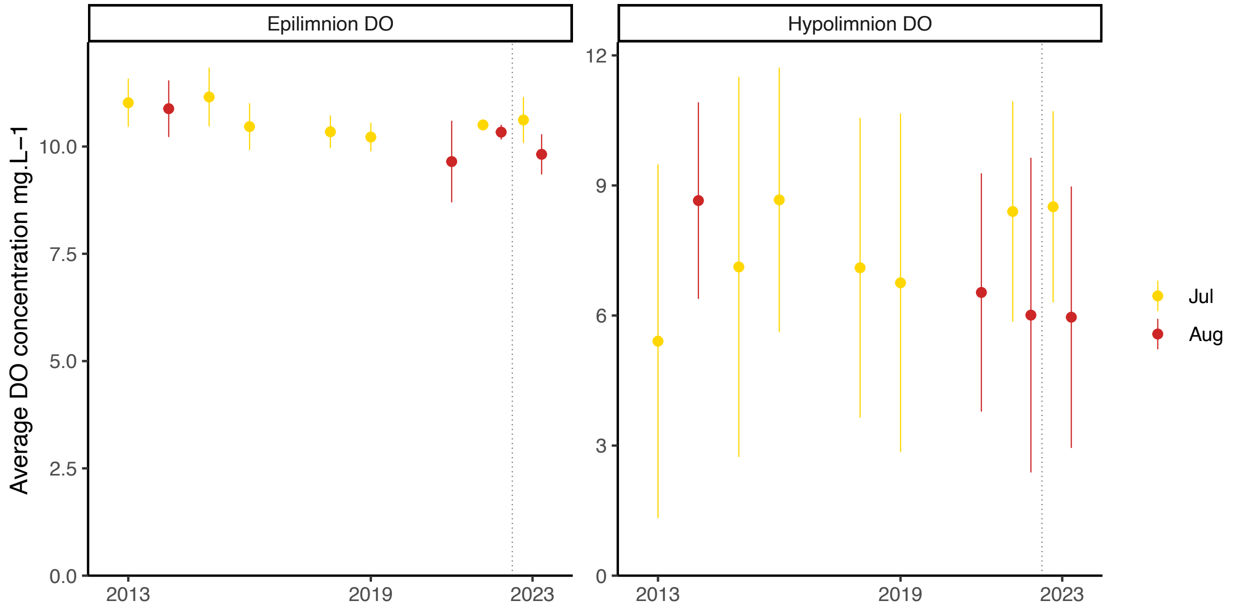

Fig. S12. Dissolved oxygen concentrations in epilimnion and hypolimnion. July (yellow) and August (red) data are compared since 2013. Central point is the median value across lakes, the line depicts the interquartile range.

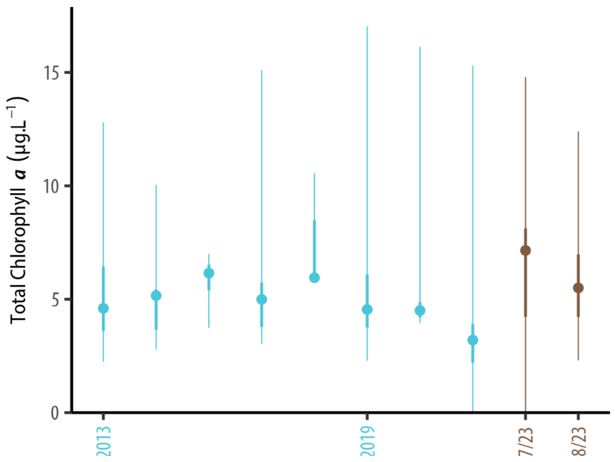

Fig. S13. Water-column integrated chlorophyll *a* concentrations. July and August 2023 (brown) data are compared to summer data (June - August) collected since 2013 (blue). Central point is the median value across lakes, the thicker line depicts the interquartile range, and the thinner line shows data within the 90th percentile.

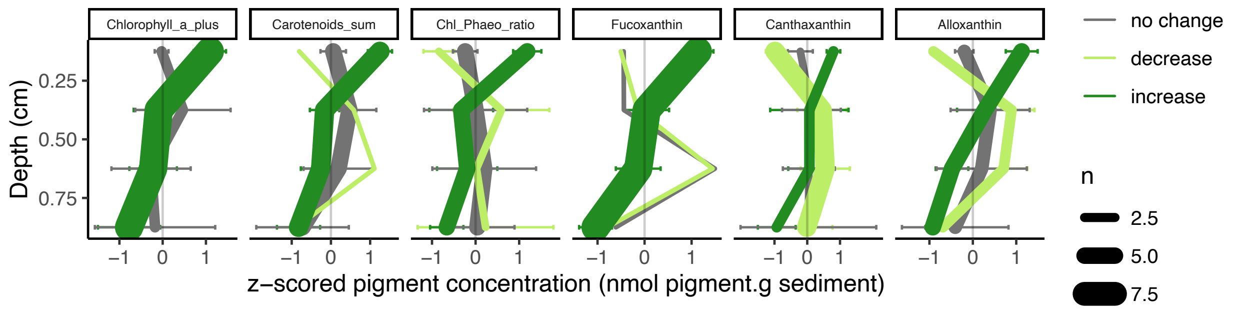

Fig. S14. Z-scored photosynthetic pigment concentrations in sediments. Pigment concentrations were measured from each lake at 0.25 cm resolution. We compare the pigment concentrations in the top sediment slice (0-0.25 cm) with average concentrations (±SD) from the three slices below (0.25 - 1.00 cm) and assess the direction of change—increase (dark green), decrease (light green), or no change (grey). The thickness of each line is proportional to the number of lakes that exhibit that particular trajectory. Line shows averages and error bars are standard deviations. We only show clusters of pigment types that are relatively stable in lake sediments.

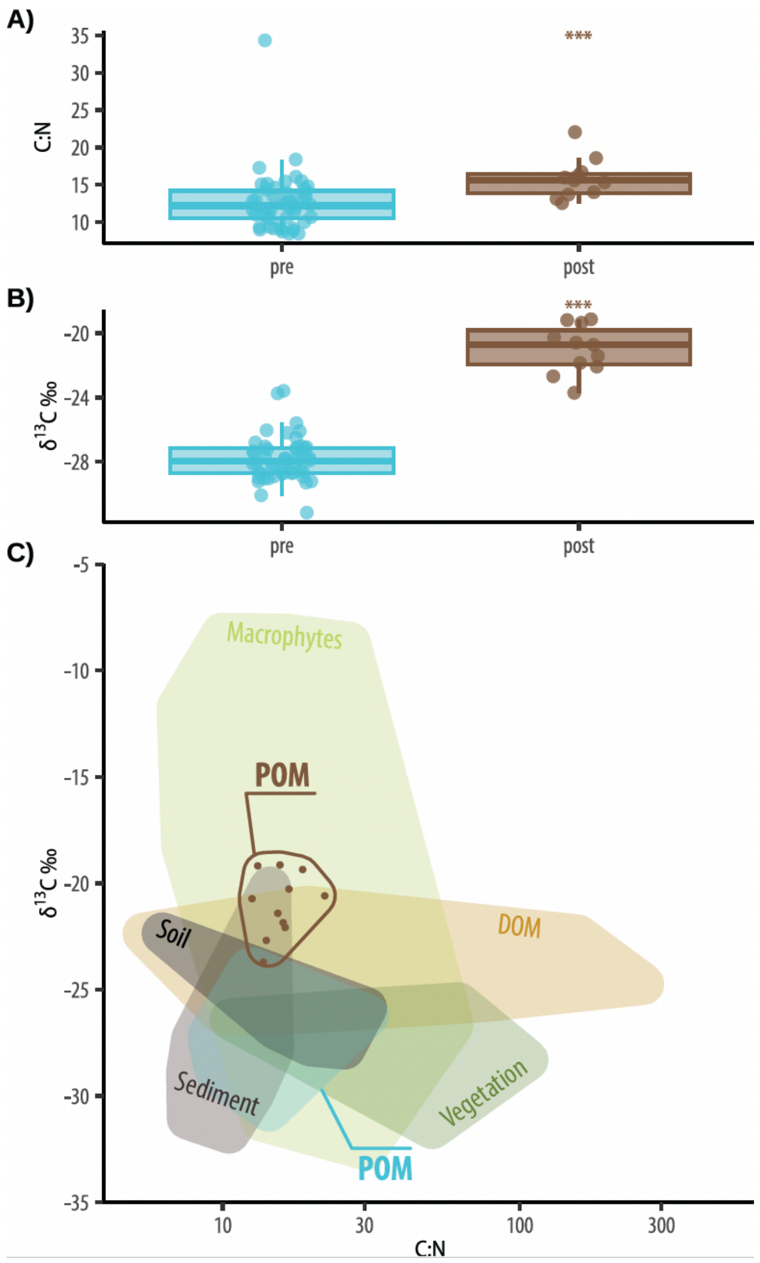

Fig. S15. Shifts in particulate organic matter (POM) A) C:N ratios, and B) $\delta^{13}C$ associated with the ecosystem transformation in 2023. Pre data were collected during years 2009 - 2014 and were reported in (*17*), post data were collected in 2023; lake names were not reported in (*17*) and therefore data are not paired. C) Biplot of C:N ratios and $\delta^{13}C$ values for sources of organic matter lakes. Hulls show signatures of sources covering both means and standard deviations, data from (*17*). Blue hull highlights POM signatures pre-browning and the brown hull with points representing measurements from each lake shows the signatures of POM in 2023.

Table S1. Carbon to nitrogen ratios (C:N) of biopolymers (BP) and humic substances (HS) in lake water dissolved organic material in two lakes pre (2018) and post (2023) September 2022.

Lake	Year	BP	HS
SS1590	2018	9.45	27.30
	2023	18.39	38.52
SS906	2018	5.83	21.57
	2023	13.99	27.31

Table S2. Rotifer feeding guild ratios (GR) in six lakes pre (2014) and post (2023) September 2022. GR is calculated as (raptorial abundance – microphagous abundance) / (total abundance). The GR ranges from -1 to +1, with GR < 0 indicating dominance by microphagous groups, and GR > 0 indicating dominance by raptorial groups.

Lake	2014	2023
SS2	-1	-0.8
SS8	-0.9	-0.7
SS85	-0.9	-1
SS903	-0.91	-1
SS1381	-0.87	-1
SS1590	-0.96	-0.96

Table S3. Planktonic consumer to producer (C:P) biomass ratios in six lakes pre (2014) and post (2023) September 2022. Ratios are based on zooplankton biomass estimates and integrated water column algal biomass based on chlorophyll *a*.

Lake	2014	2023
SS2	2.7	4.3
SS8	2.1	1.0
SS85	4.6	11.8
SS903	5.0	0.6
SS1381	3.5	3.0
SS1590	3.9	3.6

Table S4. 16sV4 primer sequences used for metabarcoding

Gene	Target	Name	Sequence 5' – 3'	Base pairs (bp)
16s V4	Prokaryotes	515 F (Parada et al. 2016)	GTGYCAGCMGCCGCGGTAA	~ 390 bp
		806 R (Apprill et al. 2015)	GGACTACNVGGGTWTCTAAT	

Movie S1. Animation of total column precipitable water standardized anomalies for September 2022 using 3-hourly time slices from reanalysis. Anomalies in reference to 1951–2000 climatology. Dataset: ECMWF Reanalysis Version 5 (ERA5) (*15*), processed using ClimateReanalyzer.org.

Movie S2. Animation of total column precipitable water standardized anomalies for October 2022 using 3-hourly time slices from reanalysis. Anomalies in reference to 1951–2000 climatology. Dataset: ECMWF Reanalysis Version 5 (ERA5) (*15*), processed using ClimateReanalyzer.org.

Movie S3. Animation of total column precipitable water standardized anomalies for July 2023 using 3-hourly time slices from reanalysis. Anomalies in reference to 1951–2000 climatology. Dataset: ECMWF Reanalysis Version 5 (ERA5) (*15*), processed using ClimateReanalyzer.org.

References

1. N. Chen, T. S. Bianchi, B. A. McKee, J. M. Bland, Historical trends of hypoxia on the Louisiana shelf: applications of pigments as biomarkers. *Organic Geochemistry* 32, 543-561 (2001).
2. J. R. Helms, A. Stubbins, J. D. Ritchie, E. C. Minor, D. J. Kieber, K. Mopper, Absorption spectral slopes and slope ratios as indicators of molecular weight, source, and photobleaching of chromophoric dissolved organic matter. *Limnology and Oceanography* 53, 955–69 (2008).
3. R. Pereira, V. N. Panizzo, J. Bischoff, S. McGowan, J. Lacey, H. Moorhouse, N. S. Zelani, M. S. Ruslan, S. Fazry, Investigating the role of hydrological connectivity on the processing of organic carbon in tropical aquatic ecosystems. *Frontiers in Earth Science* 11 (2023).
4. S. A. Huber, A. Balz, M. Abert, W. Pronk, Characterization of aquatic humic and non-humic matter with size-exclusion chromatography- organic carbon detection- organic nitrogen detection (LC-OCD-OND). *Water Research* 45, 879-885 (2011).
5. E. Morriën, S.E. Hannula, L. B. Snoek, N. R. Helmsing, H. Zweers, M. de Hollander, R. Luján Soto, M.-L. Bouffaud, M. Buée, W. Dimmers, H. Duyts, S. Geisen, M. Girlanda, R. I. Griffiths, H.-B. Jorgensen, J. Jensen, P. Plassart, D. Redecker, R. M. Schmelz, O. Schmidt, B. C. Thomson, E. Tisserant, S. Uroz, A. Winding, M. J. Bailey, M. Bonkowski, J. H. Faber, F. Martin, P. Lemanceau, W. de Boer, J. A. van Veen, W. H. van der Putten, Soil networks become more connected and take up more carbon as nature restoration progresses. *Nature Communications* 8, 14349 (2017).
6. J. G. Caporaso, G. Ackermann, A. Apprill, M. Bauer, D. Berg-Lyons, J. Betley, N. Fierer, L. Fraser, J. A. Fuhrman, J. A. Gilbert, N. Gormley, G. Humphrey, J. Huntley, J. K. Jansson, R. Knight, C. L. Lauber, C. A. Lozupone, S. McNally, D. M. Needham, S. M. Owens, A. E. Parada, R. Parsons, G. Smith, L. R. Thompson, L. Thompson, P. J. Turnbaugh, W. A. Walters, L. Weber, EMP 16S Illumina Amplicon Protocol. protocols.io https://dx.doi.org/10.17504/protocols.io.kqdg3dzzl25z/v2 (2023).
7. B. J. Callahan, P. J. McMurdie, M. J. Rosen, A. W. Han, A. J. A. Johnson, S. P. Holmes, DADA2: High-resolution sample inference from Illumina amplicon data. *Nature Methods* 13, 581-583 (2016).
8. P. J. McMurdie, S. Holmes, phyloseq: an R package for reproducible interactive analysis and graphics of microbiome census data. *PLOS One* 8, e61217 (2013).
9. S. Pighini, M. Ventura, F. Miglietta, G. Wohlfahrt, Dissolved greenhouse gas concentrations in 40 lakes in the Alpine area. *Aquatic Sciences* 80, 32 (2018).
10. R. Wanninkhof, Relationship between wind speed and gas exchange over the ocean revisited. *Limnology and Oceanography Methods* 12, 351-362 (2014).
11. B. Jähne, H. Hauβecker, Air-water gas exchange. *Annual Review of Fluid Mechanics* 30, 443–468 (1998).
12. J. J. Cole, N. F. Caraco, Atmospheric exchange of carbon dioxide in a low-wind oligotrophic lake measured by the addition of SF6. *Limnology and Oceanography* 43, 647-656 (1998).
13. D. Vachon, Y. T. Prairie, The ecosystem size and shape dependence of gas transfer velocity versus wind speed relationships in lakes. *Canadian Journal of Fisheries and Aquatic Sciences* 70, 1757-1764 (2013).
14. Z. Shen, X. Yu, Y. Sheng, J. Li, J. Luo, A Fast algorithm to estimate the deepest points of lakes for regional lake registration. *PLoS ONE*, 10: e0144700 (2015).
15. H. Hersbach, B. Bell, P. Berrisford, G. Biavati, A. Horányi, J. Muñoz Sabater, J. Nicolas, C. Peubey, R. Radu, I. Rozum, D. Schepers, A. Simmons, C. Soci, D. Dee, J.-N. Thépaut, ERA5 hourly data on single levels from 1940 to present. Copernicus Climate Change Service (C3S) Climate Data Store (CDS) (2018).
16. G. J. C. Underwood, C. L. Osburn, N. J. Anderson, M. Giles, Optical properties of chromophoric dissolved organic matter (CDOM) in arctic lakes of Southwest Greenland. [Data Collection]. Colchester, University of Essex. 10.5526/ERDR-00000067 (2017).
17. C. L. Osburn, N. J. Anderson, M. J. Leng, C. D. Barry, E. J. Whiteford, Stable isotopes reveal independent carbon pools across an Arctic hydro-climatic gradient: Implications for the fate of carbon in warmer and drier conditions. *Limnology and Oceanography Letters* 4, 205-213 (2019).
